# Theoretical Investigations on Interactions of Arylsulphonyl Indazole Derivatives as Potential Ligands of VEGFR2 Kinase

**DOI:** 10.3390/ijms21134793

**Published:** 2020-07-07

**Authors:** Kornelia Czaja, Jacek Kujawski, Paweł Śliwa, Rafał Kurczab, Radosław Kujawski, Anna Stodolna, Agnieszka Myślińska, Marek K. Bernard

**Affiliations:** 1 Chair and Department of Organic Chemistry, Faculty of Pharmacy, Poznan University of Medical Sciences, ul. Grunwaldzka 6, 60-780 Poznań, Poland; czaja.kornelia@gmail.com (K.C.); aniastodolna@gmail.com (A.S.); agnieszka.myslinska@gmail.com (A.M.); 2 Faculty of Chemical Engineering and Technology, Cracow University of Technology, ul. Warszawska 24, 31-155 Kraków, Poland; pawel.sliwa@pk.edu.pl; 3 Maj Institute of Pharmacology, Polish Academy of Sciences, ul. Smętna 12, 31-343 Kraków, Poland; kurczab@if-pan.krakow.pl; 4 Chair and Department of Pharmacology, Faculty of Pharmacy, Poznan University of Medical Sciences, ul. Rokietnicka 5a, 60-806 Poznań, Poland; radkuj@ump.edu.pl

**Keywords:** azoles, kinases, VEGFR2 kinase, DFT calculations, semi-empirical calculations, docking, PIEDA analysis, molecular dynamics, hydrogen bond

## Abstract

Vascular endothelial growth factor receptor 2 (VEGFR2) is a key receptor in the angiogenesis process. The VEGFR2 expression is upregulated in many cancers so this receptor is an important target for anticancer agents. In the present paper, we analyse interactions of several dimeric indazoles, previously investigated for anticancer activity, with the amino acids present in the VEGFR2 binding pocket. Using the docking method and MD simulations as well as theoretical computations (SAPT0, PIEDA, semi-empirical PM7), we confirmed that these azoles can efficiently bind into the kinase pocket and their poses can be stabilised by the formation of hydrogen bonds, π–π stacking, π–cation, and hybrid interactions with some amino acids of the kinase cavity like Ala866, Lys868, Glu885, Thr916, Glu917, and Phe918.

## 1. Introduction

Kinases are enzymes, which regulate protein phosphorylation and signalling pathways. Disruption of kinases function leads to many diseases including cancer. Thus, these enzymes constitute an important target for therapeutic agents [1,2]. One of the most studied types of kinases is the vascular endothelial growth factor receptor (VEGFR) family that consists of three tyrosine kinases, namely VEGFR-1, VEGFR2, and VEGFR-3. These kinases bind the angiogenesis-promoting protein—vascular endothelial growth factor (VEGF). The binding results in the activation of VEGFR and, consequently, in the formation of new blood vessels. The most important for carcinogenesis is VEGFR2, a kinase that is the major endothelial VEGF signalling receptor. Activation of VEGFR2 not only stimulates angiogenesis but also turns on other signalling pathways that include, inter alia, the PI3K-AKT-mTor pathway responsible for cell survival [3,4,5,6]. Overexpression of VEGFR2 is a typical feature of solid tumours, especially carcinomas and gliomas (bladder carcinoma, brain glioma, breast, cervical, colon, kidney, non-small celled lung, ovarian, pancreatic, and prostate cancers), which need a lattice of new blood vessels to grow beyond a 1–2 mm diameter and spread metastases [7]. However, there is evidence that angiogenesis plays a significant role in the progression of haematological malignancies as well [8,9].

**Scheme 1:**
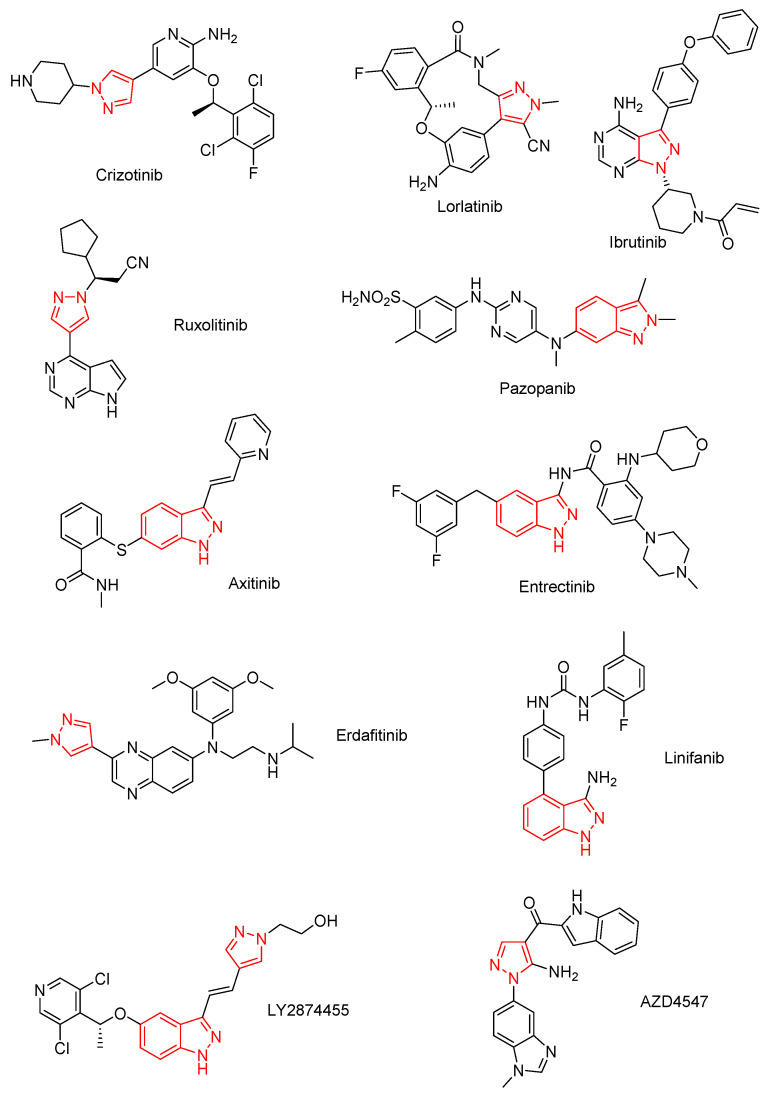
FDA-approved kinase inhibitors and examples of VEGFR2 inhibitors under clinical trials that contain pyrazole or indazole unit within the structure.

Pyrazole and indazole derivatives constitute an important group of kinase inhibitors [10,11,12,13]. Several anticancer agents containing the above heterocyclic rings have been approved for use in the treatment of solid tumours and leukemia (Scheme 1). Crizotinib is a first generation ALK and c-Met kinase dual inhibitor, approved in 2011 for the treatment of non-small cells lung cancer (NSCLC). Unfortunately, NSCLC patients rapidly developed resistance to crizotinib [14], which spurred search for new, more effective anticancer agents. Lorlatinib belongs to the third generation of ALK inhibitors. This 12-membered macrocycle containing a pyrazole scaffold was approved in 2018 for the treatment of anaplastic large cell lymphoma (ALCL) and neuroblastoma [14]. Ruxolitinib is a Janus kinase (JAK) inhibitor, the first drug that was approved for the treatment of intermediate- and high-risk myelofibrosis in 2011 [11]. Ibrutinib, a pyrazolopyrimidine derivative, is a Bruton’s tyrosine kinase (BTK) inhibitor useful for the treatment of mantle cell lymphoma and chronic lymphocytic leukemia. This anticancer agent got FDA approval in 2013 [11].

Pyrazole and indazole derivatives are among seminal VEGFR2 inhibitors (Scheme 1). Pazopanib and axitinib are small-molecule multikinase inhibitors, but they are especially effective in angiogenesis blockage through VEGFR signalling pathway inhibition. Both these drugs were approved for the therapy of metastatic renal cell carcinoma in 2009 and 2012, respectively, and Pazopanib got another FDA approval for soft tissue sarcoma in 2012 [11,15,16]. Another multitargeted indazole analogue—entrectinib, albeit is a weaker VEGFR2 inhibitor and acts mainly as a TRKA/B/C, ROS1, and ALK kinases blocking agent—was successfully examined as a promising agent for the treatment of haematologic malignancies [17,18]. Although erdafitinib is primarily a fibroblast growth factor receptor (FGFR) inhibitor, it also binds to VEGFR2 kinase [19]. Erdafitinib was approved in 2019 for the treatment of metastatic urothelial carcinoma [20].

A plethora of pyrazole and indazole derivatives are currently undergoing clinical trials. Some examples are shown in Scheme 1. Linifanib is a novel, potent ATP-competitive VEGFR/PDGFR inhibitor, effective in mutant-dependent cancer cells like FLT3, now in phase 3 clinical trials [21]. LY2874455 is a pan-FGFR and VEGFR2 inhibitor, currently in two clinical trials for the treatment of FGFR-dependent tumours [20,22]. AZD4547 is a multikinase inhibitor that binds to FGFR, VEGFR2, as well as to transmembrane tyrosine kinases CSF1R and Kit. This potential anticancer agent has undergone several clinical trials targeting NSCLC, squamous cell lung, breast, stomach, and bladder cancers, as well as lymphomas and myelomas [20].

Our previous biological investigations revealed that some indazole derivatives significantly inhibited the viability of colorectal adenocarcinoma cell line HT-29 as well as human mammary gland adenocarcinoma cell lines MCF7 and MDA-MB-231 [23,24]. The obtained results revealed that pyrazole **4** and carbazole **7** have the strongest cytotoxic activity. We then tested a hypothesis that the mechanism of the indazoles anticancer activity was related to apoptosis and caspases stimulation, but the results were somewhat inconclusive. Nevertheless, we assumed that the indazole derivatives would inhibit proteins involved in uncontrolled cell proliferation rather than interact with DNA.

## 2. Results and Discussion

### 2.1. Molecular Docking

**Figure 1:**
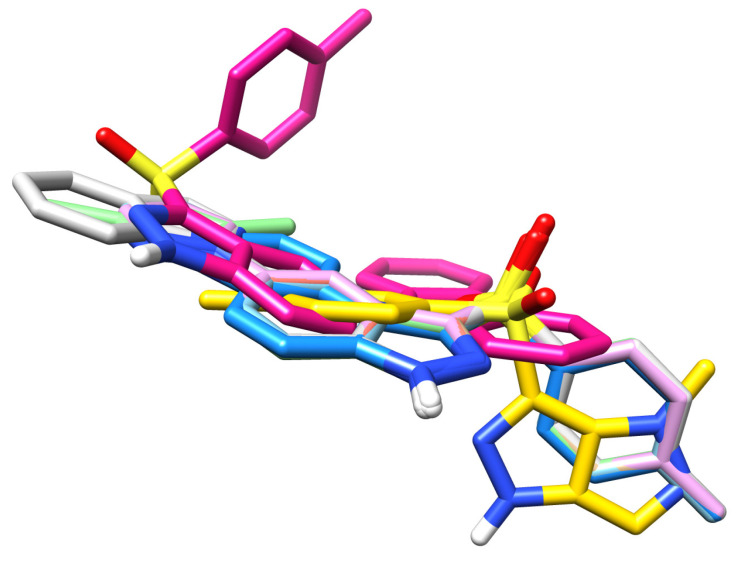
Superimposition of docked azoles **1**–**9** (first poses, *Chimera 1.13.1* package).

Nine poses were obtained for each of azole **1**–**9** from which the first poses had the lowest negative value of binding affinity (Table 1). Analysing the optimised azole ligands (*Gaussian 16 C.01* program [25]) docked to the protein (PDB code: 3ewh.pdb, *AutoDock Vina* [26]), we noticed that ligands **1**–**6** and **9** nearly overlapped (Figure 1). The carbazole ring of compound **7** was oriented almost perpendicular to the plane of the tosyl substituent on compounds **2**–**6**. On the other hand, this tosyl substituent present in compound **7** was perpendicular to the plane formed by the other heteroaromatic rings linked to the indazole moiety (compounds **2**–**6**). For fused pyrazole **8**, we observed that the planar pyrazolopyrazole core significantly superimposed with the plane of the tolyl rings of indazoles **1**–**6**, while this tolyl ring on carbazole **7** notably overlapped with the indazole plane for derivatives **1**–**6**.

Next, the molecular electrostatic potential (MEP) was determined by the B3LYP/6-311++G(2d,3p) approach for the conformers of azoles **1**–**9** (first poses) with geometry previously optimised at B3LYP/6-31G(d,p) level of theory in the gaseous phase (Figures S1–S3, Supplementary Material). In our investigations, involving the multilevel approach to the conformational rotamers search, the results were refined using a basis set enriched with higher-level polarization functions.

The obtained results show that the pyrrolic nitrogen of the pyrazole ring, an H-bond donor, and the electron-withdrawing tosyl substituent are the most important for the azole–protein interactions. Moreover, the pyridinic nitrogen of the pyrazole ring, as well as the same type of atom in the quinoline ring (compound **9**), may be of significance for each interaction. However, the contribution of these heteroatoms to the interaction strength with polar amino acids in the kinase pocket can particularly be determined by conformational factors. The stereochemical factors also influence contacts and interaction energy of the dimeric heterocycles containing an additional pyrrole, pyrazole, triazole, indole, or carbazole ring.

**Figure 2:**
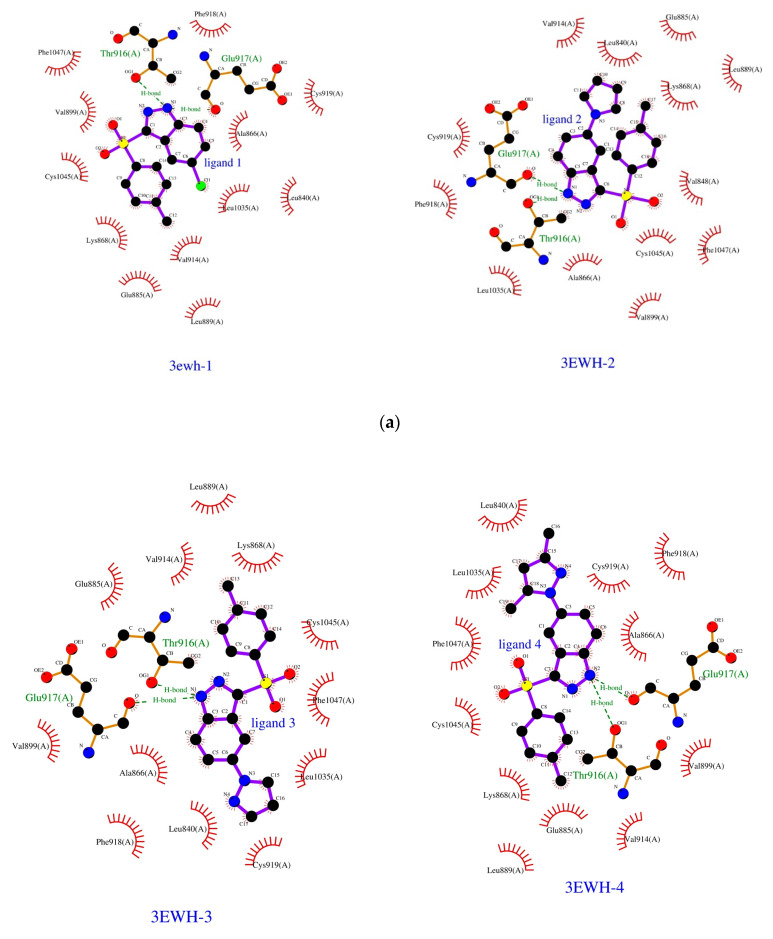

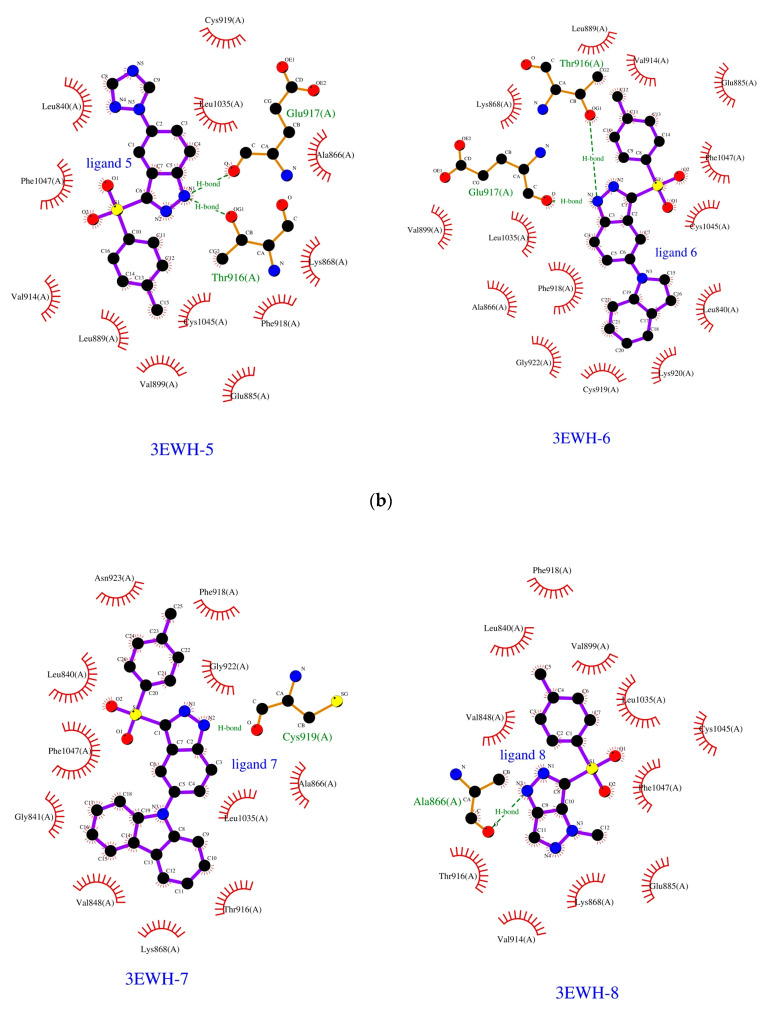

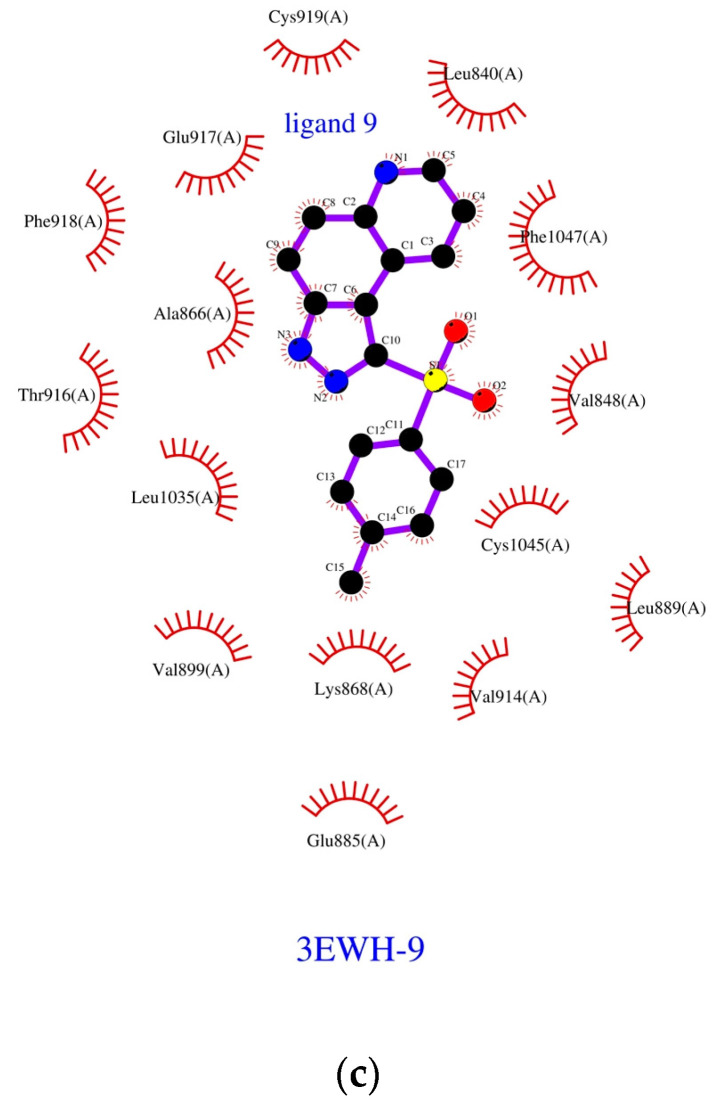
(**a**) Docking poses of indazoles **1**–**2** (first poses, *LigPlot+ v.2.2* software); (**b**) Docking poses of indazoles **3**–**6** (first poses, *LigPlot+ v.2.2* software); (**c**) Docking poses of azoles **7**–**9** (first poses, *LigPlot+ v.2.2* software).

Fitting the first poses of azoles **1**–**9** in the VEGFR2 domain by the use of a protein 3ewh.pdb [27,28] that contained K11 ligand resulted in the formation of several hydrogen bonds between the ligands and the kinase amino acids. In the docking procedure, we considered the distance *d* ≤ 3.2 Å between a proton and a heteroatom of the adjacent molecule (Table 2, Figure 2a–c). We observed that the binding modes of the docked conformers (first poses, Figure 1) of azoles **1**–**6** and **9** were almost identical. This binding pattern had particular importance for the interactions involving the polar NH and sulphonyl groups in the kinase pocket. Considering the overlap of ligands, the tosyl plane of azoles **2**–**6** and **9** was oriented nearly perpendicular to the carbazole plane (compound **7**) while their indazole rings were positioned differently despite significant superimposition. Thus, in the kinase pocket, we observed different distribution of functional groups of ligands **2**–**6** and **9** in comparison with indazole **7**.

Apart from potential anticancer agents **7** and **8**, all other azole derivatives donate an H-bond from pyrrolic nitrogens to the carbonyl of the backbone Glu917. The contact distance was in a range of 2.249–2.298 Å. The longest distance was detected for indazole derivative **3**, whereas the shortest one was noticed for analogue **9,** which included quinoline within its structure. Pathak et al. [29] observed a similar interaction involving Glu917 and series of quinazoline clubbed 1,3,5-triazine derivatives but the hydrogen bond distances were a bit longer than these described in the present paper (ca 2.56 Å). Such interaction was also reported for 5-methoxy-derivative of sunitinib and its ^11^C-radiolabeled analogue [30].

The pyrrolic NH atom was responsible for significant stabilisation of ligands **1**–**6** and **9** within the kinase cavity by forming a hydrogen contact with the Thr916 oxygen atom. The distance between the labile NH proton and H-bond acceptor ranged from 2.577 to 2.684 Å with the lowest value for indazole **2** and the highest one for quinoline derivative **9** (Table 2). The binding pose of the above azoles was furthermore stabilised by a strong hydrogen contact involving the indazole pyridinic nitrogen and the hydroxy group proton of Thr916. The distances of this contact covered a narrow range 2.103–2.170 Å and, similarly to the above contact, the shortest distance was observed for compound **2**, the longest for compound **9**. The possibility of interactions with Thr917 within the VEGFR2 cavity had been observed earlier for some benzimidazole derivatives [31] and pyridine carbonitrile analogues. [32] Similar interactions involving quinazoline clubbed 1,3,5-triazines had been reported as well, but the hydrogen contacts had been significantly longer and had ranged from 3.250 to 3.420 Å. [29].

There is a great deal of evidence that for the interactions with ligands, Cys919 is an important amino acid present in the VEGFR2 domain [29,32,33,34]. For example, it was reported that the Cys919 amide group made a hydrogen bond to the ligand for pazopanib, axitinib, and sunitinib as well as its methoxy analogue [15,16,30]. Our studies have shown that the hydrogen contact involving Cys919 can be observed only for indazole **7**. This ligand is stabilized in the kinase cavity by two hydrogen bonds, namely N-H^…^O=CCys919 and N(H)^…^H-NCys919. The distance for the latter contact is comparable with the literature data, i.e., 3.335 Å vs. 3.760 Å for one of the quinazoline clubbed 1,3,5-triazine derivatives. [29]. However, the distance between other ligands and Cys919 was greater than 4.5 Å. Such large separation excludes the probability of the existence of a hydrogen bond involving these molecules.

Similarly, a hydrogen contact N-H^…^O=C with Ala866 was detected only for fused pyrazole derivative **8**. Its distance was a little shorter (2.5 Å) than its equivalent reported for quinazoline clubbed 1,3,5-triazines [29]. For the remaining azoles, the distance to Ala866 was greater than 5.500 Å.

The participation of Glu885 in the interactions within the VEGFR2 cavity was observed for several heterocyclic ligands including pazopanib [15,29,33,34,35,36]. Although we did not notice strong hydrogen contacts that involved the carbonyl group of the above amino acid and our ligands, we spotted that the tolyl group in compounds **1**–**6** and **9** was in relatively close proximity to this functionality (2.533–2.968 Å, Table 2). This observation suggests the presence of a weak kind of hydrogen bond between this carbonyl and the tolyl methyl group and might have some implications for the stability of the ligand in the kinase domain.

The literature data show that Lys868 belongs to the kinase amino acids engaged in the interactions with several ligands. [15,31,34,36]. Analysing the geometry of the azole ligands **1**–**9** docked to VEGFR2, we found that the poses of conformers **1**–**6** and **9** located in the kinase pocket were almost identical. The distances between the ammonium group of Lys868 and the azoles sulphonyl functionality or the tosyl phenyl ring were about 4.900 Å or 4.300 Å, respectively, suggesting the presence of a π–cation contact. In comparison to ligands **1**–**6** and **9**, the tosyl phenyl ring of compound **8** was rotated nearly 180° in respect to the symmetry axis that went across the sulphur atom of the sulphone group. The above reorganisation of geometry leads to a conclusion that this contact is negligible for condensed pyrazole **8**. The distance between the tosyl group of **7** and Lys868 was also significantly extended, but here one of the carbazole benzene rings was in close proximity to the Lys868 ammonium group (ca 3.100 Å). Such distance was enough to meet the requirements for π–cation interactions. Note that a similar π–cation contact was observed for pazopanib although it involved the indazole ring [15].

It is worth noting that, apart from the hydrogen bonding, we observed π–π stacking interactions for the azoles of similar geometry, namely compounds **2**–**6** and **9**, and the phenyl ring of Phe918. The average distance involving this phenyl and the heteroaromatic dimeric rings was ca 3.400 Å. Because of a somewhat different orientation of indazole **7** in comparison to the poses of azoles **1**–**6** and **9**, the π–π stacking interactions involving compound **7** and Phe918 weakened as the distance between the arene rings extended to ca 3.600 Å. This distance was ca 4.700 Å for fused azole **8,** which had a completely different orientation within the kinase cavity. It means that the π–π stacking interactions between aromatic rings of fused azole **8** and Phe918 are practically negligible.

Such π–π stacking interactions were also detected for the Ph1047 phenyl group and heteroaromatic rings of **2**–**6** and **9**. The rings involved in the interactions were T-shaped arranged with a 3.700 Å distance separating the rings. For compound **1**, the distance between the indazole system and Ph1047 was significantly longer (ca 4 Å) than one would expect for optimal π–stacking. Albeit the compound **8** tosyl ring and Phe1047 were separated by only 2.5 Å, the reciprocal arrangement of both moieties formed a sharp angle. The azole condensed ring was too remote from the amino acid to form π–π stacking interactions. On the other hand, the tosyl ring of indazole **7** was oriented parallel with respect to Phe1047. Thus, one can assume that these systems participate in π–π stacking interactions.

Regarding the above discussion, we can conclude that indazoles **2**–**6** quinoline **9** interact with the amino acids present in the VEGFR2 pocket in a similar manner to the known drugs like pazopanib, axitinib, or sorafenib [15,16,36], i.e., by forming contacts with Cys 868, Glu885, Cys919, or Phe1047.

The above discussion leads to a conclusion that the 3-arylsulfonylindazole ligands with chlorine and small molecular azole substituent on position 5 of indazole, as well as quinoline derivative form coherent binding mode in the kinase VEGFR2 pocket. On the other hand, the presence of the pyrazolopyrazole condensed ring (compound **8**) or larger substituent like carbazole (compound **7**), results in a different pose and dissimilar distribution of hydrogen bondings in the enzyme cavity.

### 2.2. SAPT Analysis of Ligand-Amino Acid Complexes

Next, we employed the above data for the analysis of interaction energy of ligands **1**–**9** with the amino acids involved in the hydrogen bonding or π–π stackings (Table 2, Figure S12, Supplementary Material). One of the well-recognized methods is the symmetry-adapted perturbation theory (SAPT). For the analysis, we used the *Psi4 1.3.2* software [37] treating the complexes ligand-amino acid as a closed-shell system [38,39] and utilizing the recommended jun-cc-pVDZ basis set [40]. The SAPT method provides a means of directly computing the noncovalent interaction between two molecules, that is, the interaction energy is determined without computing the total energy of the monomers or dimer. In addition, SAPT provides decomposition of the interaction energy into physically meaningful components: i.e., electrostatic, exchange repulsion, induction, and dispersion terms. In SAPT, the Hamiltonian of the dimer is partitioned into contributions from each monomer and the interaction:

H = F_A_ + W_A_ + F_B_ + W_B_ + V (1)

Here, the Hamiltonian is written as a sum of the usual monomer Fock operators, F, the fluctuation potential of each monomer, W, and the interaction potential, V. The monomer Fock operators, F_A_+F_B_, are treated as the zero^th^-order Hamiltonian, and the interaction energy is evaluated through a perturbative expansion of V, W_A_, and W_B_. Through first order in V, electrostatic and exchange interactions are included; induction and dispersion first appear at second order in V [41].

The results are gathered in Table 3. The highest negative total energy SAPT0 (−6.27 kcal/mol) for the interaction with Ala866 was obtained for condensed pyrazole **8** with electrostatics, exchange, induction, and dispersion terms as follows: −5.32, 5.67, −1.48, −5.14 kcal/mol, respectively.

The interactions involving Thr916 and azoles **1**–**6** and **9** had similar energy of ca –7 kcal/mol. A similar tendency was observed for the interactions involving Glu917 with ligands **1**–**6** and **9** where the energy was about −9 kcal/mol. For both Thr916 and Glu917, the lowest energy was obtained for the interactions with indazole **5**.

For the interactions with Cys919, the lowest total energy SAPT0 was calculated for indazole **7**. The energetic components were as follows: −4.92 kcal/mol (electrostatics term), 5.80 (exchange term), −2.57 (induction term), −4.020 kcal/mol (dispersion term). A similar value of the total interaction energy was obtained for quinoline derivative **9**: 5.470 kcal/mol, with the following energetic terms: electrostatic −0.040, exchange 14.400, induction −3.110, and dispersion term −5.780 kcal/mol.

The total energy values for the interactions involving Glu855 and azoles **1**, **3**–**6** and **9** were comparable and varied from −1.660 (**5**) to −1.190 (**6**) kcal/mol, whereas these energy values for indazoles **2** and **7** were close to −1 kcal/mol. The energy value differed notably for the fused pyrazole **8**, where it equalled 10.230 kcal/mol with the following electrostatics, exchange, induction, and dispersion terms: 6.230, 16.480, −8.340 and −4.130 kcal/mol, respectively. In comparison, these terms for indazole **3** were −0.690, 2.260, −0.930, −2.310 kcal/mol, respectively.

The same analysis by the SAPT approach showed that the interaction energy involving Phe918 was the lowest for indole **6** (energetic components, i.e., electrostatics, exchange, induction, and dispersion terms were as follows: −3.830, 11.340, −1.580, −9.600 kcal/mol, respectively) and the highest for fused pyrazole **8** (energetic components, i.e., electrostatics, exchange, induction, and dispersion terms were as follows: −0.490, 2.810, −0.290, −2.860 kcal/mol, respectively), while the energy for Phe1047 was the lowest for carbazole **7** (electrostatics, exchange, induction and dispersion terms were as follows: −8.120, 19.980, −2.350, −19.460 kcal/mol, respectively) and the highest for fused azole **9** (electrostatics, exchange, induction and dispersion terms were as follows: −1.630, 12.680, −1.940, −8.040 kcal/mol, respectively).

The above findings support the conclusions drawn from the docking studies. The results confirm the presence of interactions between the azole ligands **1**–**9** and amino acids of the kinase pocket, particularly involving Ala866 (**8**), Glu885 (**1**–**6** and **9**), Thr916 (**1**–**6** and **9**), Glu917 (**1**–**6** and **9**), or Cys 919 (**7**).

### 2.3. PIEDA Analysis of Ligand-Protein Complexes

The application of quantum chemical methods for biological systems is usually computationally expensive. The fragment molecular orbital method (FMO) [42] is a convenient tool to calculate the energy of large systems at the ab initio level. The results give additional data that are troublesome to obtain with simple molecular mechanical methods. Originally, the FMO method simplified the total energy of a molecule or a molecular cluster divided into N fragments as the following sum:

(2) $$E=\sum_{I>J}E_{IJ}\;-(N-2)\sum_{I}\;E_{I}(2)$$

where E_I_, E_IJ_ are the energies of the monomer and dimer, respectively. For the receptor-ligand complexes, each residue which participates in ligand binding could be represented by a fragment, whereas ligands can be represented by single or multiple fragments as necessary. The result is the matrix of individual pair interaction energies between all fragments. Additionally, the applied pair interaction energy decomposition analysis method (PIEDA or FMO-EDA) [43] supplies the electrostatic (E_es_), exchange (E_ex_), charge transfer and mixed terms (E_CT+mix_), and dispersion (E_disp_) contributions to the total interaction energies (E_tot_), which is particularly useful for studying protein-ligand complexes. The FMO method, in its most commonly used two-body expansion (FMO2), has two steps. In the first step, the many-body polarization is accounted for by performing self-consistent quantum mechanics (QM) fragment calculations in the electrostatic field of the protein, whereas quantum effects are accounted for at the intrafragment level. This field, denoted as the electrostatic potential (ESP), is computed from the electron densities of fragments. In the second step, fragment pair calculations are performed in the converged ESP to consider interfragment quantum effects, such as: charge transfer and exchange repulsion. The FMO methodology was successfully applied to various large biological systems, primarily in a retrospective analysis of binding sites, but also as a tool supporting drug design [44]. On this account, we have focused on one of the interactions of synthesised in our group pyrazole derivatives **1**−**9** within the VEGFR2 cavity. For this purpose, we applied the polarizable continuum (PCM) solvation model [45] with water as solvent on the MP2/6–31G* level of theory using the *GAMESS* program [46].

Table 4 shows values of the total energy of interaction (TIE) for ligands **1**–**9** as a sum of interaction energies (E_tot_) of these indazole derivatives with various amino acids present in the VEGFR2 kinase pocket (pair interaction energy, E_tot_ = PIE ≥ 3 kcal/mol). The lowest value was found for indole **6** (TIE = −66.500 kcal/mol), a little higher energy was calculated for pyrrole **2**, (TIE = −61.600 kcal/mol), triazole **5** (TIE = −56.800 kcal/mol), and 3,5-dimethylopyrazole **4** (TIE = −56.200 kcal/mol). The least favourable TIE of −36.500 kcal/mol was computed for condensed pyrazole **8**.

**Figure 3:**
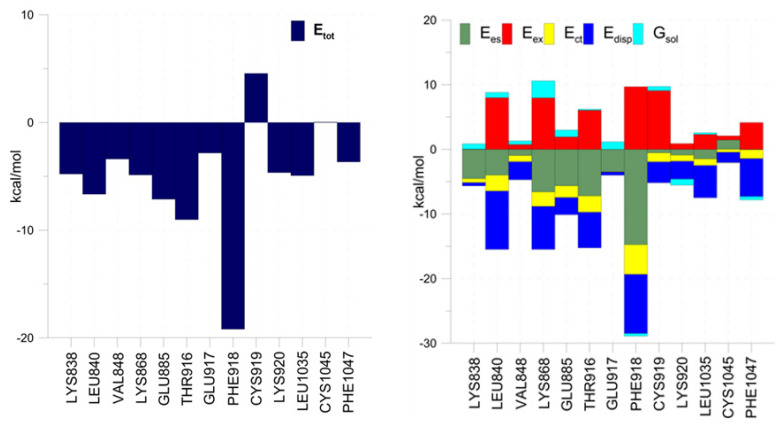
Calculated total interaction energies (E_tot_; kcal/mol) and the contributions to the total energy (E_es_, E_ex_, E_ct_ + mix, E_dis_, G_sol_; kcal/mol) between docked azole **6** and selected residues of 3ewh.pdb kinase (*GAMESS* program).

The data visualized as histograms (Table 5, Figure 3, Figures S4–S11, Supplementary Material) shows a significant level of interactions of ligands **1**–**6** and **9** with Phe918, particularly visible for indazole **5** (E_tot_ = −19.200 kcal/mol) and **6** (E_tot_ = −18.820 kcal/mol). In contrast, for indazole **7**, this parameter had a notably higher value (E_tot_ = −6.700 kcal/mol). The values for energetic terms and dispersion were variable for indazoles **6**, **5,** and **7**, namely electrostatics: −14.740, −15.850, −4.610 kcal/mol, exchange: 9.690, 9.600, 0.830 kcal/mol, and dispersion: −9.130, −7.140, −3.320 kcal/mol, respectively. It means that for indazoles **5** and **6**, the interactions with Ph918 are more polar while for carbazole **7** are more hydrophobic in nature. Note that both the SAPT (Table 3) and PIEDA approach favour indazole **6** as the best ligand for Phe918 from among the studied compounds, whereas the condensed azole **8** is the lowest in this ranking.

Next, we investigated the mutual interactions of ligands **1**–**9** with Phe1047, which was able to form π–π stackings due to the presence of the phenyl ring. Both methods revealed that carbazole **7** with a distinct dispersion term was favoured for this type of interactions (PIEDA: E_tot_ = −10.470 kcal/mol; −6.100, 15.420, −16.940 kcal/mol for electrostatics, exchange, dispersion terms, respectively). On the other hand, the π–π stacking interactions involving quinoline **9** were disfavoured (PIEDA: E_tot_ = −1.45 kcal/mol; −2.390, 10.920, −7.380 kcal/mol for electrostatics, exchange, dispersion terms, respectively), which was in accordance with the SAPT analysis.

Similar to the data from the SAPT analysis, ligands **1**–**6** and **9** interacted significantly with Thr916. The estimated energy ranged from −9.20 for **3** to −8.40 kcal/mol for **2**. The energetic terms were as follows: −7.460, 6.453, −5.710 kcal/mol for electrostatics, exchange, dispersion terms, respectively. Condensed azole **8** and indazole **7** showed the weakest interactions. For the latter compound, E_tot_ was −1.700 kcal/mol with the values for electrostatics, exchange, dispersion terms as follows: 0.090, 2.205, −2.460 kcal/mol, respectively).

The PIEDA approach, in accordance with the SAPT method, supported the relatively strong interactions of azoles **1**–**6** and **9** with Glu917. Both methods showed that indazole **5** was involved in the strongest interactions with this amino acid (PIEDA: E_tot_ = −4.870 kcal/mol; the share of the energetic components was as follows: −4.770, 0.001, −0.510 kcal/mol for electrostatics, exchange, dispersion terms, respectively), whereas the weakest interactions were observed for fused pyrazole **8** (PIEDA: E_tot_ = −0.660 kcal/mol; the share of the energetic components was as follows: −0.310, 0, −0.280 kcal/mol for electrostatics, exchange, dispersion terms, respectively). Thus, we have revealed that the potential interactions of carbazole **7** with Thr916 as well as fused pyrazole **8** with Thr916 and Glu917 are rather insignificant.

The results of the SAPT analysis concerning the interactions of azoles **1**–**9** with Ala866 were not entirely consistent with the PIEDA outcome. The PIEDA approach showed that the lowest interaction energy E_tot_ could be attributed to quinoline **9** (E_tot_ = −3.030 kcal/mol, the share of the energetic components was as follows: −0.670, 1.220, −3.220 kcal/mol for electrostatics, exchange, dispersion terms, respectively), whereas pyrazolopyrazole **8** had the highest total energy (E_tot_ = −0.220 kcal/mol, the share of the energetic components was as follows: 1.020, 2.480, −2.950 kcal/mol for electrostatics, exchange, dispersion terms, respectively). The total energy value for the latter compound leads to the conclusion that there is practically no interaction between **8** and Ala866. For condensed azoles **8** and **9**, particularly noteworthy is the relationship between electrostatics and dispersion terms.

It is interesting that for the identical change in free energy of solvation G_sol_ = 0.340 kcal/mol, the E_CT+mix_ contribution was different for compounds **8** and **9**, i.e., −1.100 for **8** and −0.690 kcal/mol for **9**. We should emphasize that the SAPT method treats the complexes ligand-amino acid as an isolated individua in the gas phase, i.e., as closed-shell systems. Considering these results, we predict that the interaction azole **8**-Ala866 is of hydrophobic character.

The disparity between the SAPT and PIEDA results can be observed for the interactions between azoles **1**–**9** and Glu885. The lowest energy E_tot_ was found for quinoline **9**: −7.52 kcal/mol; the distribution of energetics terms: −6.280, 1.730, −1.720, −2.550, 1.310 kcal/mol for E_es_, E_ex_, E_CT+mix_, E_disp_ and G_sol_, respectively. The energy calculations concerning indazole **3** gave somewhat different values for E_tot_: −4.960 kcal/mol; the energetics terms: −4.290, 0.830, −1.320, −2.070, 1.890 kcal/mol for E_es_, E_ex_, E_CT+mix_, E_disp_ and G_sol_, respectively. The dissimilarities between ligands **3** and **9** in their interactions with Glu885 can be attributed to the differences in polarity and exchange repulsion terms.

The results from the SAPT analysis for compounds **8** and **7** are reflected in the PIEDA approach, i.e., both these compounds show a poor affinity towards Glu885. The interaction energy E_tot_ for the first compound was −3.490 kcal/mol with 1.090, 4.550, −1.880, −2.670 and −2.400 kcal/mol for E_es_, E_ex_, E_CT+mix_, E_disp_ and G_sol_ terms, respectively, whereas we were not able to estimate these parameters for the second one.

Considering the interaction of azoles **1**–**9** with Cys919, both SAPT and PIEDA approach indicated carbazole **7** as a ligand with the highest negative interaction energy. The PIEDA method gave E_tot_ = −7.300 kcal/mol with −3.77,0 4.350, −2.610, −4.480, −0.790 kcal/mol for E_es_, E_ex_, E_CT+mix_, E_disp_ and G_sol_ terms, respectively. On the other hand, the weakest interaction was calculated for quinoline **9**. The PIEDA method yielded E_tot_ = 6.11 kcal/mol with −2.770, 15.290, −1.870, −4.700, 0.160 kcal/mol for E_es_, E_ex_, E_CT+mix_, E_disp_ and G_sol_ terms, respectively.

The PIEDA approach showed that the interaction with Lys838 was possible for ligands **2**–**4** and **6**, whereas for the remaining azoles, we were not able to obtain satisfactory results. The lowest energy E_tot_ was calculated for indole **6**: −4.79 kcal/mol with −4.52, 0.03, −0.63, −0.5, 0.83 kcal/mol for E_es_, E_ex_, E_CT+mix_, E_disp_ and G_sol_ terms, respectively, whereas the highest one for pyrrole analogue **2**: −3.05 kcal/mol with −1.66, 0.002, −0.08, −0.20, −1.10 kcal/mol for E_es_, E_ex_, E_CT+mix_, E_disp_ and G_sol_ terms, respectively. Significant discrepancies can be observed by comparing the values of electrostatics and charge transfer components of total energy for indazoles **2** and **6**.

The interaction of azoles **1**–**9** with Lys868, noticed during the docking procedure, was additionally proved by the PIEDA method. Here, we obtained the highest negative value E_tot_ for pyrrole **2**: −6.380 kcal/mol with −6.450, 7.770, −2.320, −6.860 and 1.470 kcal/mol for E_es_, E_ex_, E_CT+mix_, E_disp_ and G_sol_ terms, respectively. The computations provided the smallest negative value of E_tot_ for fused pyrazole **8**: −2.150 kcal/mol with −2.920, 3.370, −1.720, −5.940 and 5.060 kcal/mol for E_es_, E_ex_, E_CT+mix_, E_disp_ and G_sol_ terms, respectively. Similar to the above observation, notable dissimilarities can be observed by comparing the values of electrostatics and charge transfer components of total energy for azoles **2** and **8**.

The above discussion concerning the PIEDA analysis confirms the conclusions drawn from the docking protocol (Table 2) as well as the results of the SAPT method (Table 3). All the methods applied substantiate the hypothesis that the azole ligands can interact with the amino acids present in the VEGFR2 pocket by hydrogen bonding and π–π stacking.

### 2.4. Estimation of the Interaction Energy

In the next step, we focused on the assessment of enthalpy changes of the interactions of azole ligands **1**–**9** (Δ*H*_int_) in the VEGFR2 pocket. In this evaluation, we considered values of the final heat of formation (HOF) under standard conditions using the *Mopac 2016* program and its implemented module Mozyme [47]. To study the interactions between ligand and kinase pocket, the binding sphere was limited to 4 Å from the best pose. The pocket amino acids were correctly protonated, and the C- and N-terminal amino acids were ionised to obtain COO^−^ or NH_3_^+^. Then the hydrogen atoms of the ligand-protein complex were optimised as well as the ligand environment leaving the COO^−^ or NH_3_^+^ groups frozen. The resulted distances and polar interactions in such optimised complexes are shown in Table 6.

For the interaction energy calculations (Table 7), we adopted the approach based on the thermodynamic cycle of Raha and Merz [48]:

Δ*H*_int_ = Δ*H*_f(PL)_ − [Δ*H*_fcomplex(P)_ + Δ*H*_fcomplex(L)_] (3)

where Δ*H*_f(X)_ is the heat of formation in vacuo of the protein-ligand complex, free ligand (L) or free protein (P), and the Δ*H*_fcomplex(X)_ parameter corresponds to the enthalpy of the protein or ligand molecule in the complex conformation.

The application of the above equation to the complexes of ligands **1**–**9** with 3ewh.pdb kinase provided the values shown in Table 6 and Table 7. We analysed changes in the hydrogen contacts distribution (Table 6) that had been observed previously in the docking protocol (Table 2). The implementation of the PM7 functional for ligands **1**–**6** and **9** resulted in a distance shortening between the tosyl methyl group and the carbonyl of glutamic acid Glu885.

The semi-empirical approach also resulted in changes concerning interactions of azoles **1**–**9** with Thr917. The hydrogen bond between the indazole pyrrolic nitrogen of pyrrole **2**, 3,5-dimethylpyrazole **4**, indole **6**, as well as quinoline **9** and the oxygen atom of the Thr916 hydroxy group was slightly shortened. On the other hand, the same contact for compounds **1**, **3**, and especially **5** was lengthened. The elongation of this hydrogen contact observed for azole **5** was so sizeable (Δ = 1.602 Å) that it practically disappeared.

The same method applied to estimate the importance of the hydrogen contacts involving the indazole pyridinic nitrogen of azoles **1**–**6** and **9** and the hydroxy proton of Thr916 resulted in a significant increase of the bond lengths to 3.235–4.457 Å. Such elongation suggests that this contact is not important for the stabilisation of these azoles in the kinase pocket.

Somewhat contrasting results were obtained when the PM7 method was applied to optimisation of the hydrogen bonds connecting the ligands pyrrolic nitrogen and the Glu917 carbonyl functionality. A slight elongation of this bond was observed for pyrazole **3** and quinoline **9**. On the other hand, this bond underwent shortening for compounds **1**–**2**, **4**–**6**, particularly visible for chlorine derivative **1** (Δ = 0.471 Å). The PM7 semi-empirical calculations involving fused pyrazole **8** led to a significant extension of the N-H^…^O=CAla866 bond to 3.311 Å. This method showed that the N-H^…^OCys919 bond in carbazole **7**-Lys868 complex was shortened remarkably (Δ = 0.656 Å) implying that Cys919 was an important amino acid for the stabilisation of indazole **7** in the kinase pocket.

The next section was devoted to an analysis of the estimated enthalpy using the heat of formation values Δ*H*_f_ (Table 7) [48]. The PM7 results concerning especially azoles **7** and **9** differed from the estimated binding affinity obtained in the docking protocol, as well as the PIEDA approach (especially regarding the pyrrole derivative **2**). The greatest negative value Δ*H*_f_ was acquired for quinoline **9**. This value was almost three times greater than those for carbazole **7**, triazole **5**, or indole **6**. We should underline that the method considers only the gas phase and the possibility of the ligand relaxation inside the kinase cavity until it reaches the energy minimum. The influence of the interactions of amino acids present in the kinase pocket on the stability of the complexes ligand-protein should be verified by molecular dynamics.

### 2.5. Molecular Dynamics Calculations

**Figure 4:**
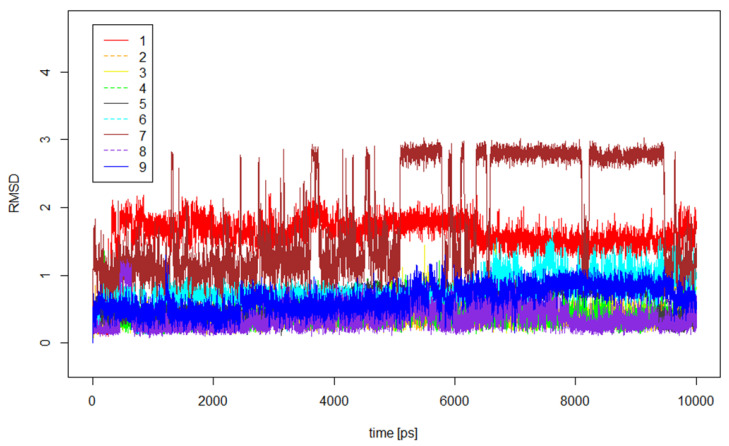
The RMSD plot for the ligand within ligand-protein complex during the MD productive phase calculated complex of kinase with **1**–**9** (*Y-*axis in Å).

Next, the 100-ns-long molecular dynamics (MD) simulations were performed to explore the stability of binding modes of ligands **1**–**9** in the VEGFR2 pocket. For this purpose, the *Desmond* software from *Schrődinger* Suite [49] was employed to simulate the solvated complexes. The time-evolution of RMSD values of the ligand in the ligand-protein complexes is shown in Figure 4.

The ligand RMSD plot shows that the docking poses of all azole ligands, apart from chlorine derivative **1**, are stable inside the kinase pocket. For most of the remaining azole ligands, the maximum trajectory deviation was running from 1.050 (**5**) to 1.650 Å (**6**). The observed RMSD fluctuations were practically analogous to the results obtained for axitinib [36], whereas for sunitinib, these values were close to 2.00 Å. For carbazole **7**, this parameter underwent changes in a 0.70–2.80 Å range at ca 50 ns.

We observed that chlorine derivative **1** had a somewhat different binding mode at the beginning of the simulation in comparison to the first framework. Such difference was due to the presence of water molecules that were taken into consideration in the MD simulation but omitted in the docking process. These water molecules not only participate in the HBs between the ligands and the protein but also form bridges between the functional groups of amino acids present in the kinase pocket. Nevertheless, the MD simulation confirmed the conclusions from the docking protocol concerning the participation of the specific amino acids in the ligand **1**–kinase interactions.

The RMSF parameter for the backbone amino acid was in a range 1.5–3.5 Å for most of azoles (Figure S21, Supplementary Material). These values correspond with the literature results [36] for axitinib and sunitinib, and demonstrate that the complexes azoles–kinase are reasonably stable. The RMSF value deviated from the above values only for indole **6**. The complex indole **6**–protein kinase reached stability in the MD simulation at about 4.5 Å. However, this situation did not lead to system destabilisation as the main contacts concerning indole **6** and the selected aminoacids were conserved (Figures S15, S18, S23–S26; Supplementary Material). Moreover, the contacts distribution involving this compound and Thr916 or Glu917 was retained as well (Figures S23–S26; Supplementary Material). Considering the structural features of azoles **1**–**9**, we should emphasise that the MD fluctuations concerned the chlorine atom (**1**), heterocyclic systems on position 5 of indazole (**2**–**7**), and tosyl ring (**1**–**9**).

**Figure 5:**
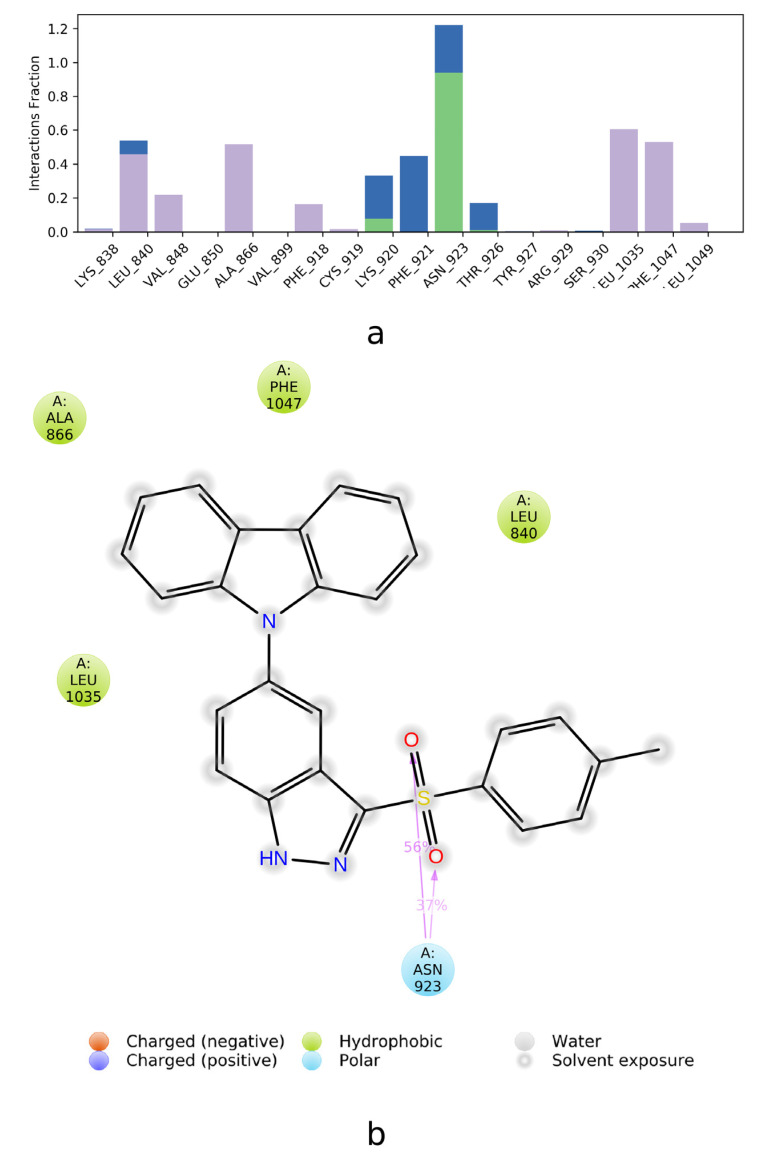
The protein-ligand interactions (**a**) hydrogen bonds—green, hydrophobic—white purple, ionic—pink, water bridges—blue) and schematic of detailed ligand atom interactions (**b**) for carbazole derivative **7** according to the MD simulations.

**Figure 6:**
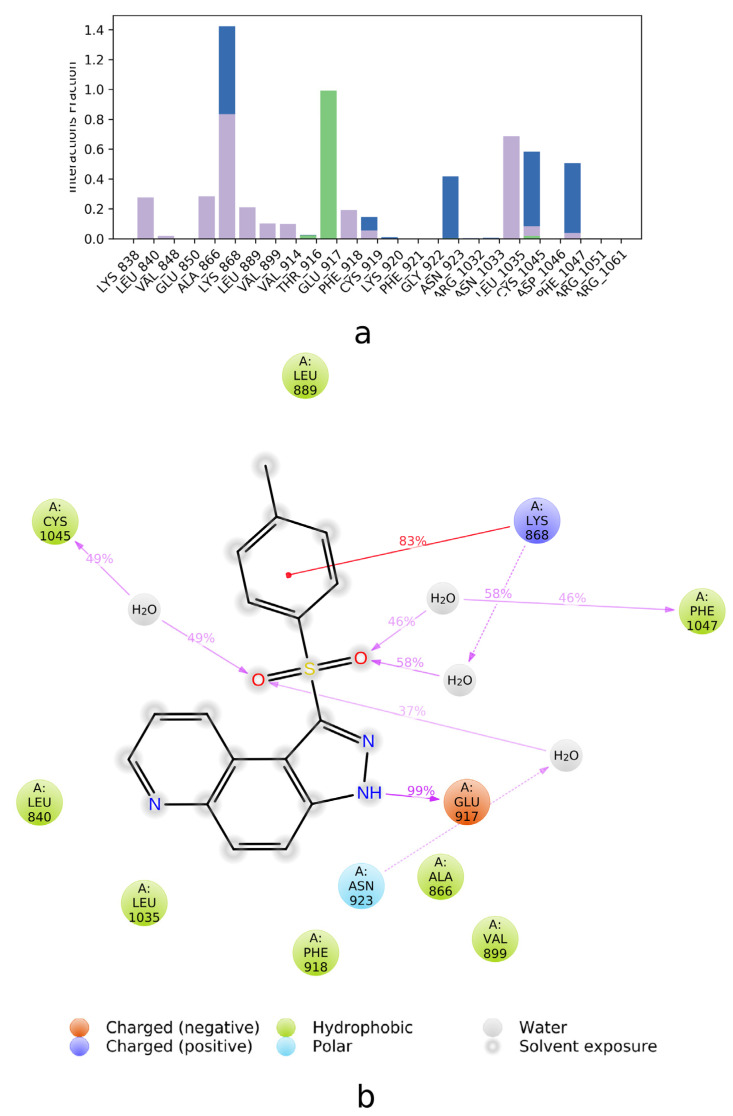
The protein-ligand interactions (**a**) hydrogen bonds—green, hydrophobic—white purple, water bridges—blue) and schematic of detailed ligand atom interactions (**b**) for quinoline derivative **9** according to the MD simulations.

Next, we decided to evaluate the distribution of contacts of azoles **1**–**9** with the amino acids present in the VEGFR2 kinase cavity, particularly those selected in the docking procedure (Table 2). The histograms and ligand-protein interactions diagrams for carbazole **7** and quinoline **9** is shown in Figure 5 and Figure 6. The graphical representations for the remaining azoles are given in Supplementary Material (Figures S13–S20).

Considering the above criteria, we observed that Glu885 interacted in the MD trajectory through water bridges only with chlorine derivative **1** and fused pyrazole **8**. The share of the sulphonyl group (compound **1**) or pyridinic nitrogen atom (compound **8**) in the interactions with water molecules was 37% or 10%, respectively. The same percentage, i.e., 37 or 10%, was observed for interactions of water bridges with Glu885.

On the other hand, the interactions between Ala866 and azoles **1**–**7** and **9** were mainly of hydrophobic character although a small percentage of hydrogen bondings and water bridges were detected for compound **1**. The sole exception was azole **8** for which the hydrogen contacts’ share was significant at 62%; the remaining interactions were of hydrophobic nature.

The hydrogen contact with Thr916, detected in the docking procedure, was also supported by the MD simulation but its share was rather small, e.g., 23% for chlorine derivative **1**, and even it was completely absent for carbazole **7**. Furthermore, it was facilitated by the contribution of water bridges for ligands **1**, **6**, and **8** as well as hydrophobic interactions for condensed azole **8**.

Considerably more intensive hydrogen contacts (99%) were observed in the MD simulation of the interactions between Glu917 and azoles **2**–**6** and **9**. For ligand **1**, this H-bond interaction had an insignificant share in comparison to the proportion of water bridges, whereas it was absent for compounds **7**–**8**.

The MD simulation concerning Phe918 and azoles **1**–**9** supports the docking data. The interactions involving Phe918 were of hydrophobic nature, although their percentages were different for the stabilisation of azole–kinase complexes. Such contact was not detected for compound **1** and had marginal values for azoles **2**–**5**. However, it was clearly visible for indole **6** as well as carbazole **7** and condensed pyrazole **8**. The hydrophobic interactions involving Cys919 and ligand **7**, observed in the docking procedure, were also detected by the MD simulation. Nevertheless, these interactions’ share was marginal for most of the azoles and imperceptible for ligand **8**. Besides, they were usually facilitated by the presence of water bridges.

The hydrophobic nature of the interactions azole ligands-Phe1047 was also confirmed by the MD simulations. Only triazole **5** was linked to the amino acid solely through water bridges. Apart from compound **7**, for which the interactions were purely hydrophobic, the remaining azoles formed with Phe1047 hybrid interactions, i.e., the hydrophobic contacts were supported by water bridges. For example, the share of water bridges in the ligands stabilisation involving the sulphone functionality was as follows: 34 (**3**), 38 (**8** the contribution of the direct interactions was 12%), or 46% (**9**).

Considering Lys838, the hydrophobic nature of its interactions with azoles **1**–**9** was supported by the MD simulations only for azoles **6** and **7** with water bridges contribution in both cases. Hybrid interactions involving Lys868 were confirmed by the MD simulations for all investigated ligands. The contribution of the sulphone group (compounds **1**, **3**, and **9**) or the pyrazole pyridinic nitrogen (compound **8**) in the interactions comprising water bridges was 48, 24, 58, or 21%, respectively, whereas the share of the direct interactions for azoles **2**, **3**, **4**, **5**, **6**, **8** and **9** was 69, 61, 63, 62, 21, 20, and 83%, respectively.

The MD simulations including Asn923, Leu1035, and Cys1045 were rather inconsistent with the docking results. The polar interaction was clearly visible for carbazole **7** (a 56% contribution), while water bridges were significant for ligands **3**–**6** and **8**–**9**. The interactions with Asn923 were not detected for azoles **1** and **2**. The MD procedure confirmed the presence of hydrophobic interactions between Leu1035 and all azoles. On the other hand, only chlorine derivative **1** interacted with Cys1045 by hydrophobic forces. For the remaining azoles, the interactions with Cys1045 involved hydrogen bonding supported by water bridges. The contribution of the sulphone group in these interactions was 92, 79, 82, 96, 62, 34, or 49% for compounds **2**, **3**, **4**, **5**, **6**, **8**, or **9**, respectively, whereas the direct hydrogen bonding share was 20, 14, 22, and 21% for azoles **2**, **3**, **5,** and **8**, respectively.

## 3. Materials and Methods

**Scheme 2:**
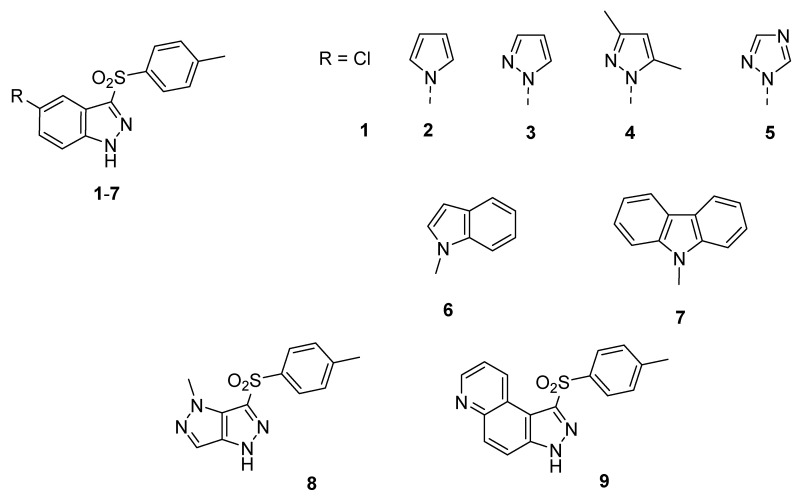
Potential VEGFR2 inhibitors studied in the present paper.

The initial preparation of the analyzed ligands **1**–**9** (Scheme 2) was carried out as described in our previous papers related with heterocyclic potential Chk1 ligands obtained in our group [50]. Next, all the resulting conformations were optimized with PM7 (*Mopac 2016*) [47,51], then each from the four most energetically stable conformers of hetarenes **1**–**9**, i.e., with the lowest HOF, was optimized using density functional theory formalism [52] in the gaseous phase. On this account, DFT calculations were executed, and geometries of each previously pre-optimized conformers of **1**–**9** (Scheme 1) were further optimized using the *Gaussian 16 C.01* program [25] at the B3LYP/6-31G(d,p) level of theory (very tight criteria) [53]. The energy minimum was confirmed by the frequency calculations for all conformers, no negative (imaginary) frequencies were detected in the generated vibrational spectrum of the analyzed conformers. The vibrational frequencies (IR spectra) and thermodynamic properties were computed using the same level of theory as for the SCF (optimization) procedure and applying the ideal gas, rigid rotor, and harmonic oscillator approximations. The molecular electrostatic potential (MEP) was determined by the B3LYP/6-311++G(2d,3p) approach for the conformers of azoles **1**–**9** (1st poses) with geometry previously optimized at B3LYP/6-31G(d,p) level of theory in the gaseous phase (*Gaussian 16 C.01* program [25], key-word, pop = esp”). The QM calculations were carried out using resources provided by the Wrocław Center for Networking and Supercomputing (*Bem* cluster).

The human VEGFR2 kinase protein in complex with a K11 derivative, acquired from the Protein Data Bank base (PDB entry: 3ewh with the resolution of 1.60 Å), was selected as the biological target [27,28] as one of the most used for docking PDB version of the human VEGFR2 kinase [29,30,31,32,33,34,35,36]. An initial target for further optimization was prepared by removing the internal ligand (K11 derivative) from the 3ewh.pdb file but keeping the internal coordinates unchanged. The genetic algorithm (GA) method implemented in the program *AutoDock Vina* [26] was employed to locate the appropriate binding orientations and conformations of the compounds into the VEGFR2 binding pocket. For each type of atom within the structure of protein or ligand, Gasteiger charges were computed. Moreover, an ‘autodock type’ was assigned to each atom. All water molecules and internal ligand (K11 as a fused azole derivative) were removed from the original PDB file (the 3ewh.pdb). Polar hydrogen atoms were added, and partial charges were assigned to the protein. Then the internal ligand was replaced by the optimized structure of investigated hetarenes **1**–**9** and additionally, the residues were saturated with hydrogen atoms. To carry out docking simulation, a grid box was defined to be of 10 Å size (centre_x = 17.783, centre_y = −7.351, centre_z = 5.179). The outputs (*.*pdbqt* files) after docking procedure were visualized using the *Chimera 1.13.1* package [54]. The projections of the 1st poses of azoles **1**–**9** docked to the kinase pocket were visualised with *LigPlot+ v.2.2* software [55,56] (Figure 2a–c).

For semi-empirical calculations with the use of the PM7 method [48], we used the *Mopac 2016* software [47] and Mozyme method [50].

On account of FMO methodology, we have focused on one of the interactions of synthesised in our group pyrazole derivatives **1**–**9** within the VEGFR2 cavity. For this purpose, we applied the MP2/6–31G* level using the *GAMESS* program [46], as well as the polarizable continuum (PCM) solvation model [45] and water as a solvent.

For molecular dynamics MD calculations, the *Desmond* software [49] was employed to simulate the solvated complexes. The OPLS3e force field [57] was used to parameterize the protein and counterions as well as ligands and their topology. Finally, the complexes were inserted into the cubic water boxes using TIP4P water model [58] (10 × 10 × 10 nm). The soluble complex consisted of one molecule of VEGFR2 kinase, one ligand molecule, approximately 37.3 k water molecules, and about 20 Na^+^ ions depending on the charge of the ligand. The soluble complexes were first minimized using the steepest descent scheme. The minimized configurations were then relaxed in NVT and NPT ensembles with 500 ps MD length per simulations. The complexes were restrained by NVT simulations using a small harmonic force and free of restraints by NPT MD simulations. The relaxed system was then used as an initial conformation of MD simulations during 100 000 ps. To this purpose, we utilised Nose-Hoover thermostat [59] with relaxation time of 1 ps at T = 300 K and Martyna-Tobias-Klein (MTTK) barostat [60] with relaxation time of 2 ps. Ligand RMSD (right *Y*-axis) indicates how stable the ligand is with respect to the protein and its binding pocket. ‘Lig fit Lig’ shows the RMSD of a ligand that is aligned and measured just on its reference conformation (zero framework). These RMSD values measure the internal fluctuations of the ligand atoms. The current geometric criteria for protein-ligand H-bonds were as follows: distance of 2.5 Å between the donor and acceptor atoms (D—H···A); a donor angle of 120° between the donor-hydrogen–acceptor atoms (D—H···A); and an acceptor angle of 90° between the hydrogen-acceptor-bonded atoms (H···A—X). The output trajectory of indazole 1 was hierarchically clustered, basing on the RMSD matrix, into 15 clusters using trajectory analysis tools from the *Maestro* (*Schrödinger*) suite. Each cluster included a representative frame (i.e., ligand–protein complex) used in further comparative analysis as is given in Figure S22 (Supplementary Material).

In some cases, hydrogen-bonded protein-ligand interactions were mediated by a water molecule. The hydrogen-bond geometries were therefore slightly relaxed from the standard H-bond definition. On this account, the current geometric criteria for the resulted water-bridges were as follows: a distance of 2.8 Å between the donor and acceptor atoms (D—H···A), a donor angle of 110° between the donor-hydrogen-acceptor atoms (D—H···A), and an acceptor angle of 90° between the hydrogen-acceptor-bonded atoms (H···A—X). Hydrophobic contacts fall into three subtypes: π–cation; π–π; and other, non-specific interactions. Generally, this type of interactions involves a hydrophobic amino acid and an aromatic or aliphatic group on the ligand, but we have extended this category to include π–cation interactions as well. The current geometric criteria for hydrophobic interactions were then as follows: π–cation for aromatic and charged groups within 4.5 Å, π–π—for aromatic groups stacked face-to-face or face-to-edge, other—related with non-specific hydrophobic sidechain within 3.6 Å of a ligand’s aromatic or aliphatic carbons. Ionic interactions or polar interactions were calculated between two oppositely charged atoms that are within 3.7 Å of each other and do not involve a hydrogen bond.

## 4. Conclusions

In the present paper, we provided evidence that derivatives of indazole and condensed pyrazole can represent valuable template hits for the VEGFR2 inhibitors.

The poses of the docked conformers of azoles **1**–**6** and **9** were practically superimposable (Table 1 and Table 2). However, as the phenyl plane of tosyl group for ligands **2**–**6** and **9** was almost perpendicular to the carbazole **7** plane, the indazole ring of compound **7** was oriented differently in comparison with the same indazole ring in the remaining azoles. Thus, even though the indazole ring in all the above ligands fitted the same plane, the dissimilar orientation of this ring in carbazole **7** resulted in a different distribution of the functional groups participating in the interactions with the amino acids of VEGFR2 pocket. A crucial role in the stability of ligands **1**–**6** and **9** in the kinase cavity was attributed to the indazole pyrrolic nitrogen, which formed a hydrogen bond with Thr916. These azoles were additionally stabilised by the hydrogen contact between the indazole pyridinic nitrogen and the hydroxy proton of Thr916.

Although we did not observe typical strong hydrogen contacts with the Glu885 carbonyl group, the methyl group of the tolyl substituent on compounds **1**–**6** and **9** was in close proximity to that carbonyl which suggests the probability of a weak hydrogen bond between these functionalities. We also observed π–π stacking interactions involving aromatic rings of azoles **2**–**6** and **9** and the phenyl residue of Phe918.

The above findings from the docking procedure were generally confirmed by the SAPT0, PIEDA, and semi-empirical PM7 methods. The calculated total interaction energy for the azole–VEGFR2 complexes ranged from −36.500 (compound **8**) to −66.500 kcal/mol (compound **6**), whereas the semi-empirical PM7 calculations of the interaction energy involving enthalpy change fluctuated from −65.830 (azole **3**) to −323.470 kcal/mol (quinoline **7**).

The molecular dynamics simulations indicate that almost all ligand–kinase VEGFR2 complexes, apart from compound **1**, presented stable binding mode. We discussed the nature of the ligand–amino acid interactions within the VEGFR2 cavity in the function of time, as well as possible formation of pure hydrogen bonds, hydrogen contacts supported by water bridges, and hydrophobic interactions.

As mentioned in the Introduction, the anticancer activity of the azoles **1**–**9** was tested in vitro on HT-29 (IC_50_ of 29.9 ± 3.5 μM for **1** and 37.3 ± 2.0 μM for **9**), MCF7 (IC_50_ of 39.7 ± 5.8 μM for **3** as an example), and MDA-MB-231 (IC_50_ of 17.7 ± 2.7 μM for **1** as an example) cancer cell lines. These tests showed that pyrazole **3** and carbazole **7** (not a surprise!), as well as indole **6** had the highest activity against the cancer cells, whereas chlorine derivative **1** and fused pyrazole **8** were the weakest cytotoxic agents (the latter compound even stimulated proliferation of the MDA-MB-231 cells) [23,24]. The theoretical studies involving docking, MD simulations, and semi-empirical calculations, reported in the present paper, confirmed the outcome of the cytotoxic tests. However, carbazole **7** is insoluble in water and polar solvents, and scarcely soluble in organic solvents. Considering the above results, we hope that indazoles **3** and **6** will constitute lead compounds for further structural modifications directed towards better potency, selectivity, and ADME properties. These structural modifications may involve the benzene part of indazole ring, replacement of sulphone fragment, and substitution at the indazole pyrrolic nitrogen.

## Figures and Tables

**Table 1:** Estimated binding affinity [kcal/mol] for the first two poses of azoles **1**–**9** generated during the docking procedure.

*Pose*	Estimated Binding Affinity of the Docked Azoles 1–9								
	Ligand 1	Ligand 2	Ligand 3	Ligand 4	Ligand 5	Ligand 6	Ligand 7	Ligand 8	Ligand 9
*1*	−8.900	−9.700	−9.600	−10.300	−9.500	−10.900	−11.600	−8.000	−9.900
*2*	−8.600	−8.900	−9.100	−10.000	−8.400	−10.300	−11.100	−7.700	−9.400

**Table 2:** Ligand-amino acid contacts (under *d* ≤ 3.2 Å) for the first poses of azoles **1**–**9** generated during the docking procedure.

HB	Contacts Calculated for Docked Azoles 1–9								
	Ligand 1	Ligand 2	Ligand 3	Ligand 4	Ligand 5	Ligand 6	Ligand 7	Ligand 8	Ligand 9
N-H^…^O=C_Ala866_	×	×	×	×	×	×	×	2.377	×
CH^…+^H_3_N_Lys868_	×	×	×	×	×	×	2.110	×	×
CH_3_^…^O=C_Glu885_	2.932	2.587	2.968	2.877	2.943	×	×	×	2.533
N-H^…^O_Thr916_	2.607	2.577	2.604	2.619	2.608	2.656	×	×	2.684
N_pyridinic_^…^H-O_Thr916_	2.166	2.103	2.158	2.147	2.159	2.165	×	×	2.170
N-H^…^O=C_Glu917_	2.274	2.266	2.249	2.263	2.255	2.263	×	×	2.298
N-H^…^O_Cys919_	×	×	×	×	×	×	2.067	×	×

**Table 3:** Calculated total values of the interaction ligand-amino acid energy [kcal/mol] using the SAPT0 method for docked azoles **1**–**9**.

Amino Acid	Calculated Value of Total SAPT0 Energy for Docked Azoles 1–9								
	Ligand 1	Ligand 2	Ligand 3	Ligand 4	Ligand 5	Ligand 6	Ligand 7	Ligand 8	Ligand 9
Ala866	−3.350	−3.700	−3.350	−3.550	−3.400	−3.400	−2.270	−6.270	−3.830
Glu885	−1.450	−0.780	−1.660	−1.400	−1.500	−1.190	0.200	10.230	−0.820
Thr916	−7.760	−6.980	−7.740	−7.330	−7.870	−7.690	−0.640	−5.240	−7.200
Glu917	−9.250	−9.160	−9.090	−8.820	−9.380	−8.980	−2.630	−0.840	−9.030
Phe918	−1.110	−2.210	−1.890	−2.080	−1.810	−3.680	−2.470	−0.840	−1.310
Cys919	2.740	2.020	3.940	3.750	1.640	1.040	−5.700	−0.690	5.470
Phe1047	−0.830	−1.560	−1.160	−0.300	−1.210	−1.280	−9.950	−1.330	1.060

**Table 4:** Calculated total values of the interactions ligand-amino acid energy (TIE) [kcal/mol] for docked azoles **1**–**9** using the PIEDA method.

Ligand	Calculated Value of Total Tie Energy for Docked Azoles 1–9								
	Ligand 1	Ligand 2	Ligand 3	Ligand 4	Ligand 5	Ligand 6	Ligand 7	Ligand 8	Ligand 9
**TIE**	−42.900	−61.600	−49.300	−56.200	−56.800	−66.500	−45.700	−36.500	−48.400

**Table 5:** Calculated total values of interaction ligand-amino acid energy (E_tot_) [kcal/mol] using the PIEDA method for docked azoles **1**–**9** (nd—no data retrieved).

Amino Acid	Calculated Value of E_tot_ Energy for Docked Azoles 1–9								
	Ligand 1	Ligand 2	Ligand 3	Ligand 4	Ligand 5	Ligand 6	Ligand 7	Ligand 8	Ligand 9
Lys838	nd	−3.050	−3.090	−4.640	nd	−4.790	nd	nd	nd
Ala866	−2.270	−2.780	−2.670	−3.000	−2.120	−2.120	−1.680	−0.220	−3.030
Lys868	−3.910	−6.380	−5.460	−6.050	−3.370	−4.870	−4.770	−2.150	−4.850
Glu885	−6.930	−6.410	−4.960	−5.460	−7.170	−7.170	nd	−3.490	−7.520
Thr916	−8.940	−8.400	−9.200	−8.890	−9.000	−9.000	−1.700	−3.600	−8.640
Glu917	−3.970	−2.200	−1.690	−1.390	−4.870	−4.870	−1.210	−0.660	−2.720
Phe918	−17.160	−17.790	−16.700	−16.540	−18.820	−19.200	−6.700	−1.720	−15.910
Cys919	4.930	3.730	4.760	4.770	4.400	4.400	−7.300	−0.650	6.110
Phe1047	−3.210	−4.390	−3.410	−2.670	−3.620	3.620	−10.470	−3.900	−1.450

**Table 6:** Ligand-amino acid distances between azoles **1**–**9** and residues around 4 Å after optimisation with the PM7 method; RMSD_complex_ = 1.455 (**1**), 0.882 (**2**), 0.893 (**3**), 0.951 (**4**), 1.216 (**5**), 0.780 (**6**), 0.942 (**7**), 1.083 (**8**) and 1.754 (**9**) Å, respectively.

Contacts	Contacts Length Calculated for Optimized Azoles 1–9								
	Ligand 1	Ligand 2	Ligand 3	Ligand 4	Ligand 5	Ligand 6	Ligand 7	Ligand 8	Ligand 9
N-H^…^O=C_Ala866_	×	×	×	×	×	×	×	3.311	×
CH^…+^H_3_N_Lys868_	×	×	×	×	×	×	2.645	×	×
CH_3_^…^O=C_Glu885_	2.377	2.107	2.130	2.443	2.313	×	×	×	2.213
N-H^…^O_Thr916_	3.710	2.498	2.909	2.512	4.258	2326	×	×	2.284
N_pyridinic_^…^H-O_Thr916_	4.213	3.841	3.879	3.097	4.457	2.609	×	×	3.235
N-H^…^O=C_Glu917_	1.803	2.058	2.258	2.235	1.917	2.072	×	×	2.487
N-H^…^O_Cys919_	×	×	×	×	×	×	1.411	×	×

**Table 7:** Calculated heat of formations [kcal/mol] for the free ligands (Δ*H*_fcomplex(L)_), free protein (Δ*H*_fcomplex(P)_), ligand-protein complex (Δ*H*_f(PL)_), as well as ligand-protein interaction energy (Δ*H*_int_).

Compound	HOF of Ligand (Δ*H*_fcomplex(L)_)	HOF of Protein (Δ*H*_fcomplex(P)_)	HOF of Complex (Δ*H*_f(PL)_)	Δ*H*_int_
**1**	−0.240	−2438.770	−2532.830	−93.810
**2**	41.550	−2117.010	−2167.680	−92.220
**3**	129.410	−1993.880	−1930.300	−65.830
**4**	42.660	−2 353.080	−2 403.600	−93.190
**5**	69.860	−2154.820	−2199.390	−114.430
**6**	57.370	−2264.260	−2308.590	−101.710
**7**	75.330	−1875.310	−1923.270	−123.290
**8**	43.520	−1562.010	−1607.010	−88.510
**9**	30.330	−3622.170	−3915.320	−323.470

## Supplementary Materials

Supplementary materials can be found at https://www.mdpi.com/1422-0067/21/13/4793/s1. Cartesian coordinates of all discussed ligands and adducts, MEP and PIEDA analysis visualization, as well as results of MD simulations are given in the Supplementary file. This information is available via the Internet or upon request from the corresponding author.
