# Supplementary material for: Theoretical Investigations on Interactions of Arylsulphonyl Indazole Derivatives as Potential Ligands of VEGFR2 Kinase
Source: Int J Mol Sci. 2020 Jul 7;21(13):4793. doi: 10.3390/ijms21134793 (PMC7369845; doi:10.3390/ijms21134793)

Supporting Information

Theoretical investigations on interactions of arylsulphonyl indazole derivatives as potential ligands of VEGFR2 kinase

Kornelia Czaja [1], Jacek Kujawski [1]*, Paweł Śliwa [2], Rafał Kurczab [3], Radosław Kujawski [4], Anna Stodolna [1], Agnieszka Myślińska [1] and Marek K. Bernard [1]*
[1]Chair and Department of Organic Chemistry, Faculty of Pharmacy, Poznan University of Medical Sciences, ul. Grunwaldzka 6, 60-780 Poznań, Poland
[2]Cracow University of Technology, Faculty of Chemical Engineering and Technology, ul. Warszawska 24, 31-155 Kraków, Poland
[3]Maj Institute of Pharmacology, Polish Academy of Sciences, ul. Smętna 12, 31-343 Kraków, Poland
[4]Chair and Department of Pharmacology, Faculty of Pharmacy, Poznan University of Medical Sciences, ul. Rokietnicka 5a, 60-806 Poznań, Poland
* Correspondence: mbernard@ump.edu.pl (M.K.B.), jacekkuj@ump.edu.pl (J.K.), phone 48618546670, fax 48618546680

Table of Contents:

Fig. S1 Electrostatic potential (ESP) map of docked azole **5** (1st pose) calculated at the B3LYP/6-311++G(2d,3p)//B3LYP-631G(d,p) level of theory (gaseous phase); isovalue = 0.002 a.u. 4

Fig. S2 Electrostatic potential (ESP) map of docked azole **7** (1st pose) calculated at the B3LYP/6-311++G(2d,3p)//B3LYP-631G(d,p) level of theory (gaseous phase); isovalue = 0.002 a.u. 4

Fig. S3: Geometry of the first poses of azoles **1**–**9** after docking procedure to 3ewh.pdb protein and their Cartesian coordinates (charge=0, multiplicity=1): 5

Fig. S4 Calculated interaction energies (E_{tot}; kcal/mol) and the contributions to the total energy (E_{es}, E_{ex}, E_{ct} + mix, E_{dis}, G_{sol}; kcal/mol) between docked azole **1** and selected residues of 3ewh.pdb kinase(*GAMESS* program). 13

Fig. S5 Calculated interaction energies (E_{tot}; kcal/mol) and the contributions to the total energy (E_{es}, E_{ex}, E_{ct} + mix, E_{dis}, G_{sol}; kcal/mol) between docked azole **2** and selected residues of 3ewh.pdb kinase(*GAMESS* program). 13

Fig. S6 Calculated interaction energies (E_{tot}; kcal/mol) and the contributions to the total energy (E_{es}, E_{ex}, E_{ct} + mix, E_{dis}, G_{sol}; kcal/mol) between docked azole **3** and selected residues of 3ewh.pdb kinase(*GAMESS* program). 14

Fig. S7 Calculated interaction energies (E_{tot}; kcal/mol) and the contributions to the total energy (E_{es}, E_{ex}, E_{ct} + mix, E_{dis}, G_{sol}; kcal/mol) between docked azole **4** and selected residues of 3ewh.pdb kinase(*GAMESS* program). 14

Fig. S8 Calculated interaction energies (E_{tot}; kcal/mol) and the contributions to the total energy (E_{es}, E_{ex}, E_{ct} + mix, E_{dis}, G_{sol}; kcal/mol) between docked azole **5** and selected residues of 3ewh.pdb kinase(*GAMESS* program). 15

Fig. S9 Calculated interaction energies (E_{tot}; kcal/mol) and the contributions to the total energy (E_{es}, E_{ex}, E_{ct+mix}, E_{dis}, G_{sol}; kcal/mol) between docked azole **7** and selected residues of 3ewh.pdb kinase(*GAMESS* program). 15

Fig. S10 Calculated interaction energies (E_{tot}; kcal/mol) and the contributions to the total energy (E_{es}, E_{ex}, E_{ct+mix}, E_{dis}, G_{sol}; kcal/mol) between docked azole **8** and selected residues of 3ewh.pdb kinase(*GAMESS* program). 16

Fig. S11 Calculated interaction energies (E_{tot}; kcal/mol) and the contributions to the total energy (E_{es}, E_{ex}, E_{ct+mix}, E_{dis}, G_{sol}; kcal/mol) between docked azole **9** and selected residues of 3ewh.pdb kinase(*GAMESS* program). 16

Fig. S12 Example of input file for SAPt calculations regarding the **1**–Glu917 complex: 17

Fig. S13 The protein–ligand interactions (**a;** hydrogen bonds – green, hydrophobic – white purple, ionic – pink, water bridges – blue) and schematic of detailed ligand atom interactions (**b**) for chlorine derivative **1**. 19

Fig. S14 The protein–ligand interactions (**a;** hydrogen bonds – green, hydrophobic – white purple, ionic – pink, water bridges – blue) and schematic of detailed ligand atom interactions (**b**) for pyrrole derivative **2**. 20

Fig. S15 The protein–ligand interactions (**a;** hydrogen bonds – green, hydrophobic – white purple, ionic – pink, water bridges – blue) and schematic of detailed ligand atom interactions (**b**) for pyrazole derivative **3** 21

Fig. S16 The protein–ligand interactions (**a;** hydrogen bonds – green, hydrophobic – white purple, ionic – pink, water bridges – blue) and schematic of detailed ligand atom interactions (**b**) for dimethyl pyrazole derivative **4**. 22

Fig. S17 The protein–ligand interactions (**a;** hydrogen bonds – green, hydrophobic – white purple, ionic – pink, water bridges – blue) and schematic of detailed ligand atom interactions (**b**) for triazole derivative **5**. 23

Fig. S18 The protein–ligand interactions (**a;** hydrogen bonds – green, hydrophobic – white purple, ionic – pink, water bridges – blue) and schematic of detailed ligand atom interactions (**b**) for indole derivative **6**. 24

Fig. S19 The protein–ligand interactions (**a;** hydrogen bonds – green, hydrophobic – white purple, ionic – pink, water bridges – blue) and schematic of detailed ligand atom interactions (**b**) for carbazole derivative **8**. 25

Fig. S20 Atoms numbering for RMSF calculations of the ligand **1** and the RMSF plot for **1** within ligand–protein complex during the MD productive phase calculated complex of kinase with **1**. 26

Fig. S21 The RMSD plot for the backbone within ligand-protein complex during the MD productive phase calculated complex of kinase with: **1**–**9** (*Y*-axis in Å); colors related with **Fig. 4** given in the manuscript. 27

Fig. S22 Representative MD cluster for binding mode of **1** within the **1**–3ewh complex (**A**) and its comparison with results using docking protocol (**B**). 28

Fig. S23 Hydrogen contacts with Thr916 during the MD productive phase calculated complex of kinase with derivative **3**; red – angles [°], blue – distances [Å]. 28
Fig. S24 Hydrogen contacts with Glu917 during the MD productive phase calculated complex of kinase with derivative **3**; red – angles [°], blue – distances [Å]. 29
Fig. S25 Hydrogen contacts with Thr916 during the MD productive phase calculated complex of kinase with derivative **6**; red – angles [°], blue – distances [Å]. 29
Fig. S26 Hydrogen contacts with Glu917 during the MD productive phase calculated complex of kinase with derivative **6**; red – angles [°], blue – distances [Å]. 30

Fig. S1 Electrostatic potential (ESP) map of docked azole **5** (1st pose) calculated at the B3LYP/6-311++G(2d,3p)//B3LYP-631G(d,p) level of theory (gaseous phase); isovalue = 0.002 a.u.

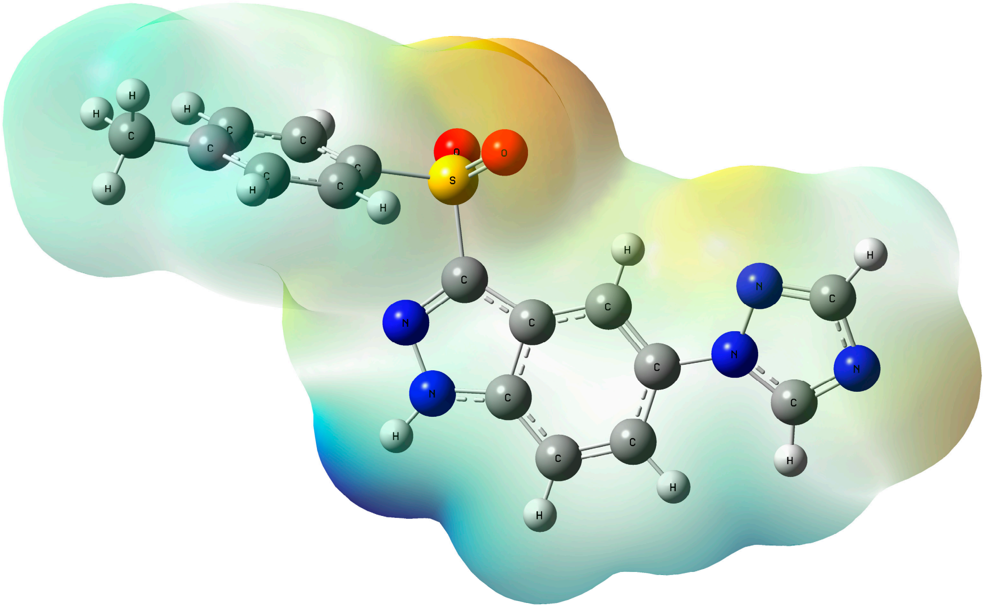

Fig. S2 Electrostatic potential (ESP) map of docked azole **7** (1st pose) calculated at the B3LYP/6-311++G(2d,3p)//B3LYP-631G(d,p) level of theory (gaseous phase); isovalue = 0.002 a.u.

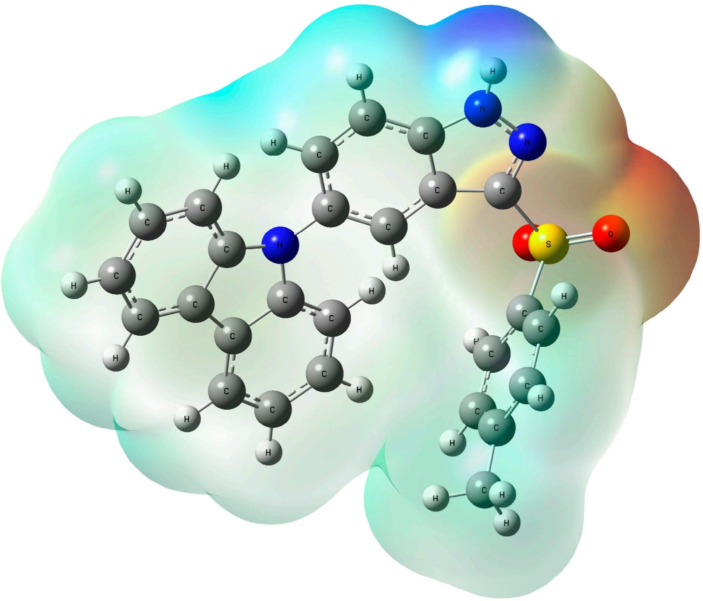

Electrostatic potential (ESP) map of ligands **1–4**, **6**, **8–9** are given in the our previous reports:

Czaja K., Kujawski J., Kamel K., Bernard M.K. Selected arylsulphonyl pyrazole derivatives as potential Chk1 kinase ligands – computational investigations. *J. Mol. Model.* **2020**, *accepted*. doi: 10.1007/s00894-020-04407-3.

Fig. S3: Geometry of the first poses of azoles **1**–**9** after docking procedure to 3ewh.pdb protein and their Cartesian coordinates (charge=0, multiplicity=1):

Azole 1

Cartesian coordinates:

C	16.051000000	-6.986000000	7.723000000
C	16.267000000	-7.422000000	6.376000000
C	15.464000000	-8.585000000	6.273000000
N	14.861000000	-8.735000000	7.496000000
N	15.217000000	-7.777000000	8.374000000
C	15.387000000	-9.338000000	5.092000000
C	16.137000000	-8.898000000	4.015000000
C	16.940000000	-7.737000000	4.116000000
C	17.028000000	-6.987000000	5.275000000
H	14.227000000	-9.457000000	7.797000000
Cl	17.851000000	-7.251000000	2.718000000
S	16.782000000	-5.579000000	8.557000000
O	15.773000000	-4.511000000	8.458000000
O	18.145000000	-5.394000000	8.045000000
C	16.896000000	-6.080000000	10.275000000
C	16.053000000	-5.490000000	11.216000000
C	16.154000000	-5.878000000	12.551000000
C	17.084000000	-6.845000000	12.956000000
C	17.209000000	-7.237000000	14.407000000
C	17.919000000	-7.420000000	11.985000000
C	17.836000000	-7.044000000	10.648000000
H	14.770000000	-10.222000000	5.026000000
H	16.109000000	-9.448000000	3.086000000
H	17.652000000	-6.108000000	5.332000000
H	15.333000000	-4.744000000	10.915000000
H	15.504000000	-5.426000000	13.286000000
H	18.639000000	-8.168000000	12.281000000
H	18.487000000	-7.490000000	9.911000000
H	16.478000000	-6.684000000	14.997000000
H	18.213000000	-7.004000000	14.761000000
H	17.026000000	-8.306000000	14.511000000

Azole 2

Cartesian coordinates:

C	17.03500	-7.04800	5.32300
C	16.92400	-7.75100	4.15000
C	16.06100	-8.88300	4.05600
C	15.32600	-9.31800	5.11900
C	15.43900	-8.58800	6.31900
N	14.84800	-8.75400	7.57000

H	14.15800	-9.45200	7.85900
N	15.25100	-7.77200	8.46700
C	16.09700	-7.02300	7.78600
C	16.26600	-7.47400	6.42300
N	17.67800	-7.35300	2.98300
C	18.67200	-6.37000	2.93200
C	19.11800	-6.27900	1.66300
C	18.39200	-7.23000	0.87500
C	17.52500	-7.86400	1.69000
S	16.86700	-5.57500	8.51400
O	18.59400	-5.55200	7.84300
O	15.60300	-4.20700	8.45400
C	16.99200	-6.07100	10.24500
C	16.17100	-5.47100	11.18800
C	16.25600	-5.87100	12.51100
C	17.14900	-6.86700	12.90000
C	17.95600	-7.46300	11.93400
C	17.25200	-7.28700	14.36400
C	17.88000	-7.07200	10.60600
H	17.69200	-6.19400	5.40000
H	15.98800	-9.41000	3.11600
H	14.68500	-10.18400	5.04400
H	19.02300	-5.78300	3.76800
H	19.88500	-5.60900	1.30500
H	18.51700	-7.40700	-0.18300
H	16.83000	-8.63600	1.39400
H	15.47300	-4.70100	10.89400
H	15.62200	-5.40500	13.25100
H	18.64900	-8.23900	12.22300
H	18.50500	-7.54200	9.86200
H	18.00000	-8.07300	14.46500
H	17.54400	-6.42800	14.96800
H	16.28600	-7.65900	14.70400

Azole 3

Cartesian coordinates:

C	16.03800	-7.00000	7.70700
C	16.24500	-7.42800	6.35600
C	15.44900	-8.59400	6.25700
N	14.86300	-8.75700	7.48600
N	15.21900	-7.80000	8.36600
C	15.36700	-9.33300	5.06600
C	16.10500	-8.88500	3.98900
C	16.90700	-7.71600	4.08000
C	16.97800	-6.97300	5.24700
H	14.23600	-9.48600	7.78900
S	16.77700	-5.59700	8.53800
O	18.14100	-5.41500	8.02800
O	15.77400	-4.52100	8.43800
C	16.88800	-6.09400	10.25900

C	16.02000	-5.52800	11.19100
C	16.11800	-5.91400	12.52700
C	17.07200	-6.85300	12.94100
C	17.93200	-7.40300	11.97900
C	17.19400	-7.24200	14.39400
C	17.85200	-7.02900	10.64000
N	17.64900	-7.32400	2.93500
C	18.62700	-6.37300	2.84800
C	19.04600	-6.34700	1.53600
C	18.26000	-7.33200	0.89400
N	17.41700	-7.92300	1.73500
H	14.75100	-10.21700	4.99500
H	16.07200	-9.43400	3.05900
H	17.57600	-6.07500	5.30200
H	15.28300	-4.80100	10.88300
H	15.44700	-5.48200	13.25500
H	18.67000	-8.13000	12.28300
H	18.52400	-7.45500	9.90900
H	19.00000	-5.75900	3.65400
H	19.80400	-5.71600	1.09600
H	18.33600	-7.57500	-0.15600
H	16.44200	-6.71100	14.97700
H	18.18800	-6.98000	14.75800
H	17.04100	-8.31600	14.49700

Azole 4

Cartesian coordinates:

C	17.04700	-7.07600	5.28200
C	16.26900	-7.48200	6.38100
C	16.94000	-7.80700	4.10800
C	15.43500	-8.62100	6.28400
C	16.10000	-8.95200	4.02600
C	15.34900	-9.37700	5.10300
C	16.06300	-7.03300	7.72400
N	15.20700	-7.79600	8.38100
N	14.83500	-8.75500	7.51000
H	14.17000	-9.45000	7.80800
S	16.78000	-5.60400	8.52700
O	18.14400	-5.49500	7.97300
O	15.83200	-4.48800	8.43600
C	16.91800	-6.07500	10.25200
C	16.08900	-5.46800	11.19600
C	16.19300	-5.85100	12.53200
C	17.11600	-6.82500	12.93700
C	17.24400	-7.21200	14.39000
C	17.93100	-7.42200	11.96300
C	17.84000	-7.05600	10.62400
N	17.67700	-7.44800	2.95000
N	17.44100	-8.13700	1.79200
C	18.22900	-7.57400	0.88200

C	18.24800	-8.08700	-0.52500
C	18.97400	-6.50700	1.44500
C	18.60000	-6.44000	2.77200
C	19.02600	-5.46600	3.82600
H	17.70600	-6.22300	5.35000
H	16.04900	-9.50200	3.09800
H	14.72200	-10.25400	5.04100
H	15.37800	-4.71300	10.89600
H	15.55100	-5.38900	13.26800
H	18.64200	-8.17900	12.25800
H	18.47200	-7.52300	9.88400
H	19.68900	-5.87500	0.93900
H	18.95100	-7.50100	-1.11700
H	18.55600	-9.13300	-0.52600
H	17.25100	-8.00200	-0.95600
H	19.79000	-4.80300	3.42100
H	18.16600	-4.87700	4.14500
H	19.43200	-6.00900	4.68000
H	16.53000	-6.64100	14.98300
H	18.25500	-6.99800	14.73500
H	17.04000	-8.27700	14.50200

Azole 5

Cartesian coordinates:

C	16.98300	-6.97900	5.25600
C	16.90800	-7.72700	4.09200
C	16.10300	-8.89100	3.99600
C	15.36000	-9.33500	5.07300
C	15.44500	-8.59400	6.26200
N	14.85500	-8.75000	7.49000
H	14.22300	-9.47500	7.79300
N	15.21300	-7.79400	8.36900
C	16.03900	-6.99800	7.71300
C	16.24600	-7.43000	6.36300
N	17.65800	-7.33800	2.94900
N	18.63000	-6.38300	3.02900
C	19.06400	-6.31700	1.78100
N	18.44400	-7.15300	0.89900
C	17.56800	-7.77600	1.66300
S	16.78700	-5.59700	8.54200
O	18.15000	-5.42500	8.02600
O	15.78700	-4.51900	8.44000
C	16.89900	-6.09300	10.26200
C	17.84700	-7.04600	10.64000
C	17.92900	-7.41700	11.97800
C	17.08400	-6.84900	12.94400
C	16.14500	-5.89300	12.53400
C	17.20700	-7.23800	14.39700
C	16.04600	-5.50800	11.19800
H	17.58500	-6.08400	5.31000

H	16.07000	-9.43900	3.06600
H	14.73900	-10.21600	5.00300
H	19.85600	-5.64700	1.48100
H	16.87600	-8.52900	1.31700
H	18.50500	-7.48700	9.90600
H	18.65700	-8.15600	12.27900
H	15.48700	-5.44700	13.26500
H	15.32100	-4.76900	10.89200
H	16.46800	-6.69200	14.98400
H	18.20700	-6.99400	14.75500
H	17.03500	-8.30900	14.50200

Azole 6

Cartesian coordinates:

C	16.06000	-6.99700	7.72800
C	16.28200	-7.40700	6.37400
C	15.46900	-8.56100	6.24400
N	14.86800	-8.73800	7.46400
N	15.21800	-7.79500	8.36100
C	15.39200	-9.28300	5.04400
C	16.15700	-8.82700	3.98600
C	16.96600	-7.66200	4.09300
C	17.02900	-6.94000	5.27900
H	14.22200	-9.45800	7.74500
S	16.73900	-5.57000	8.57100
O	18.10500	-5.42500	8.03700
O	15.76600	-4.47400	8.48600
C	16.86400	-6.07100	10.28900
C	17.82100	-7.01800	10.65500
C	17.91900	-7.39400	11.99300
C	17.07700	-6.84000	12.96600
C	17.21000	-7.23000	14.41800
C	16.12500	-5.88900	12.56500
C	16.01300	-5.49800	11.23500
N	17.70100	-7.24800	2.95400
C	18.72700	-6.30500	2.97500
C	19.22100	-6.11500	1.71700
C	18.47600	-6.97100	0.83600
C	18.50700	-7.20300	-0.55000
C	17.53100	-7.67100	1.63500
C	17.60500	-8.10200	-1.10300
C	16.66500	-8.77200	-0.29700
C	16.61100	-8.56700	1.07700
H	14.76300	-10.15600	4.95000
H	16.14000	-9.37000	3.05300
H	17.63300	-6.04800	5.35700
H	18.47500	-7.45200	9.91300
H	18.65700	-8.12600	12.28500
H	15.46800	-5.45300	13.30300
H	15.27900	-4.76300	10.93900

H	19.08100	-5.79700	3.86000
H	20.02200	-5.44700	1.43600
H	19.22200	-6.68900	-1.17600
H	17.62500	-8.29000	-2.16600
H	15.97100	-9.46000	-0.75600
H	15.88600	-9.08000	1.69100
H	17.99800	-7.97600	14.52400
H	17.46200	-6.34900	15.00900
H	16.26600	-7.64600	14.77000

Azole 7

Cartesian coordinates:

C	17.39000	-7.13500	1.97600
N	16.52300	-7.95000	1.35600
N	15.78900	-8.59300	2.29400
H	15.08800	-9.24400	2.01200
C	16.17200	-8.20300	3.57800
C	15.72000	-8.59900	4.84600
C	16.32100	-8.02300	5.94500
C	17.35600	-7.06400	5.78100
C	17.80400	-6.66400	4.53100
C	17.21800	-7.23700	3.40300
N	17.93300	-6.48800	6.94200
C	17.22000	-6.29700	8.14700
C	16.09800	-6.97300	8.62200
C	15.62500	-6.62100	9.88100
C	16.25600	-5.63200	10.64900
C	17.38400	-4.96500	10.18100
C	17.87300	-5.28700	8.92200
C	18.98100	-4.80100	8.13100
C	19.94900	-3.83000	8.34800
C	20.88100	-3.58200	7.34400
C	20.85100	-4.29100	6.13500
C	19.89700	-5.27400	5.89100
C	18.96900	-5.52800	6.89900
S	18.53100	-6.15400	1.03400
O	18.05900	-6.13400	-0.33300
O	19.86300	-6.60000	1.36600
C	18.29900	-4.54700	1.71400
C	19.30200	-3.96800	2.49100
C	19.17300	-2.65100	2.92200
C	18.03200	-1.91000	2.58800
C	17.00500	-2.51600	1.86100
C	17.93400	-0.48100	2.99000
C	17.13300	-3.83500	1.43500
H	14.93100	-9.32800	4.95700
H	16.00200	-8.30300	6.93800
H	18.58700	-5.92700	4.43400
H	15.61500	-7.74000	8.03500
H	14.75300	-7.12000	10.27600

H	15.85900	-5.38300	11.62200
H	17.86900	-4.21200	10.78500
H	19.97800	-3.27900	9.27600
H	21.64100	-2.83000	7.49900
H	21.58600	-4.06900	5.37500
H	19.87700	-5.81800	4.95800
H	20.17800	-4.54100	2.75800
H	19.95400	-2.19900	3.51500
H	16.10900	-1.96000	1.62900
H	16.33000	-4.30700	0.88900
H	16.98400	-0.07100	2.64700
H	18.75500	0.07900	2.54200
H	17.99200	-0.40400	4.07600

Azole 8

Cartesian coordinates:

O	14.76600	-6.12700	8.96700
S	16.22000	-6.00400	8.74400
O	16.89000	-4.70000	8.82700
C	16.59500	-6.72200	7.14300
C	17.79200	-6.38800	6.50800
C	18.06800	-6.93900	5.26000
C	17.16500	-7.81600	4.63700
C	17.48800	-8.42600	3.29500
C	15.96700	-8.12100	5.29600
C	15.67400	-7.58200	6.54800
C	16.97000	-7.10900	9.93000
N	17.63600	-8.17700	9.49200
N	18.11700	-8.80300	10.58400
H	18.66100	-9.64000	10.45900
C	17.77600	-8.15000	11.73800
C	17.02100	-7.03700	11.35100
C	17.85700	-8.09700	13.14800
N	16.69400	-6.37500	12.49900
N	17.19500	-7.02500	13.58100
C	15.87000	-5.19200	12.69800
H	18.49300	-5.71300	6.97700
H	18.99300	-6.68800	4.76200
H	15.25800	-8.78600	4.82600
H	14.74900	-7.82700	7.04800
H	18.37300	-8.81100	13.77400
H	16.66800	-9.07300	2.98300
H	18.40300	-9.01200	3.37400
H	17.62600	-7.63400	2.55900
H	15.55600	-4.80100	11.73000
H	14.99100	-5.45600	13.28600
H	16.44600	-4.43300	13.22700

Azole 9

Cartesian coordinates:

C	17.09300	-7.24300	5.16600
C	16.85800	-8.01100	3.98100
C	18.06800	-6.22400	5.09800
N	17.52000	-7.78500	2.81500
C	18.72800	-6.00900	3.90500
C	18.41600	-6.81300	2.79000
C	16.31400	-7.57800	6.32700
C	15.38900	-8.63800	6.25100
C	15.89800	-9.08000	3.97300
C	15.16800	-9.40700	5.07900
C	16.16600	-7.12000	7.67700
N	15.25600	-7.81100	8.34800
N	14.79800	-8.72900	7.47700
H	14.09400	-9.38300	7.78500
S	16.91800	-5.71300	8.50600
O	18.30500	-5.61200	8.01400
O	16.00600	-4.56700	8.36700
C	16.97800	-6.18700	10.23100
C	17.89900	-7.15300	10.63900
C	17.97100	-7.48700	11.98900
C	17.13900	-6.86900	12.93400
C	17.20500	-7.25800	14.39100
C	16.23200	-5.89200	12.49400
C	16.14500	-5.54600	11.14800
H	18.29400	-5.62200	5.96600
H	19.47500	-5.23300	3.82700
H	18.93800	-6.62500	1.86400
H	15.74900	-9.64300	3.06300
H	14.45300	-10.21600	5.06300
H	18.54500	-7.63400	9.91900
H	18.68000	-8.23500	12.31400
H	15.59100	-5.40100	13.21100
H	15.44400	-4.79300	10.81900
H	17.96600	-8.02600	14.52800
H	16.23700	-7.64600	14.70800
H	17.45900	-6.38300	14.98900

Fig. S4 Calculated interaction energies (E_{tot}; kcal/mol) and the contributions to the total energy (E_{es}, E_{ex}, E_{ct} + mix, E_{dis}, G_{sol}; kcal/mol) between docked azole **1** and selected residues of 3ewh.pdb kinase(*GAMESS* program).

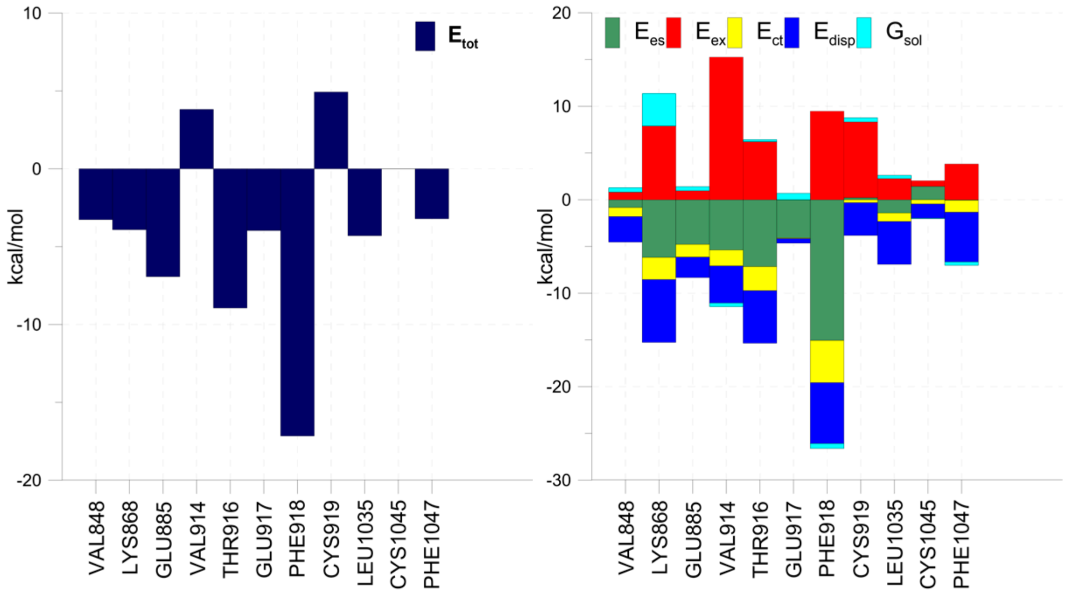

Fig. S5 Calculated interaction energies (E_{tot}; kcal/mol) and the contributions to the total energy (E_{es}, E_{ex}, E_{ct} + mix, E_{dis}, G_{sol}; kcal/mol) between docked azole **2** and selected residues of 3ewh.pdb kinase(*GAMESS* program).

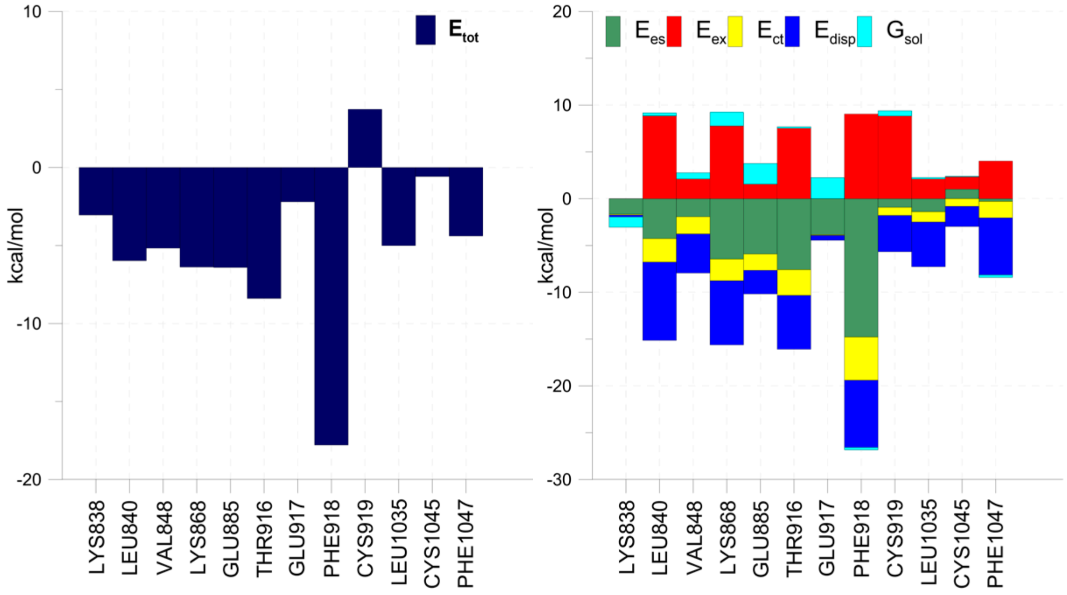

Fig. S6 Calculated interaction energies (E_{tot}; kcal/mol) and the contributions to the total energy (E_{es}, E_{ex}, E_{ct} + mix, E_{dis}, G_{sol}; kcal/mol) between docked azole **3** and selected residues of 3ewh.pdb kinase(*GAMESS* program).

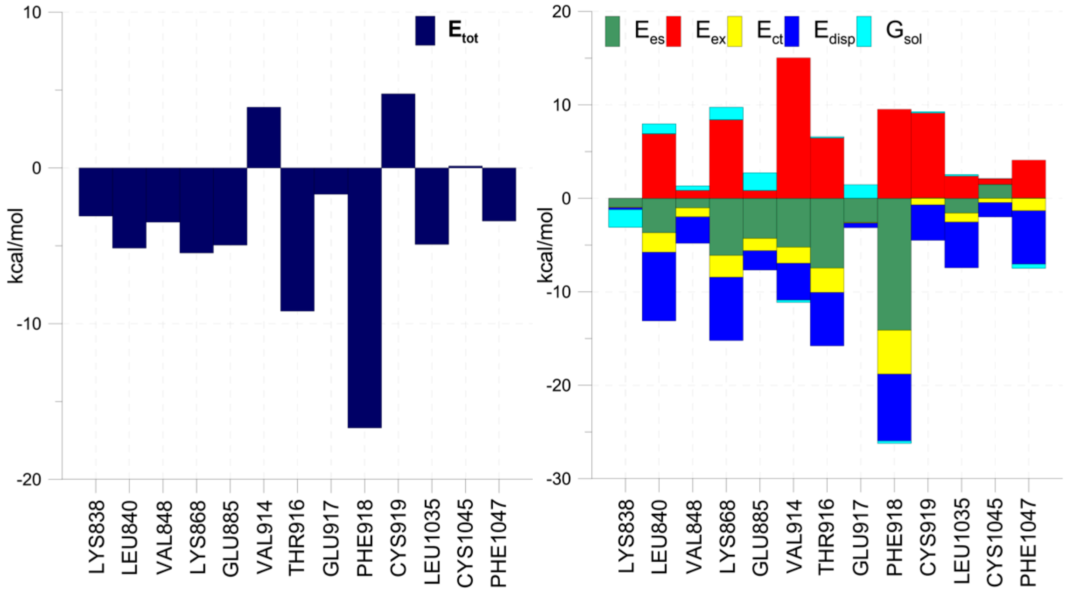

Fig. S7 Calculated interaction energies (E_{tot}; kcal/mol) and the contributions to the total energy (E_{es}, E_{ex}, E_{ct} + mix, E_{dis}, G_{sol}; kcal/mol) between docked azole **4** and selected residues of 3ewh.pdb kinase(*GAMESS* program).

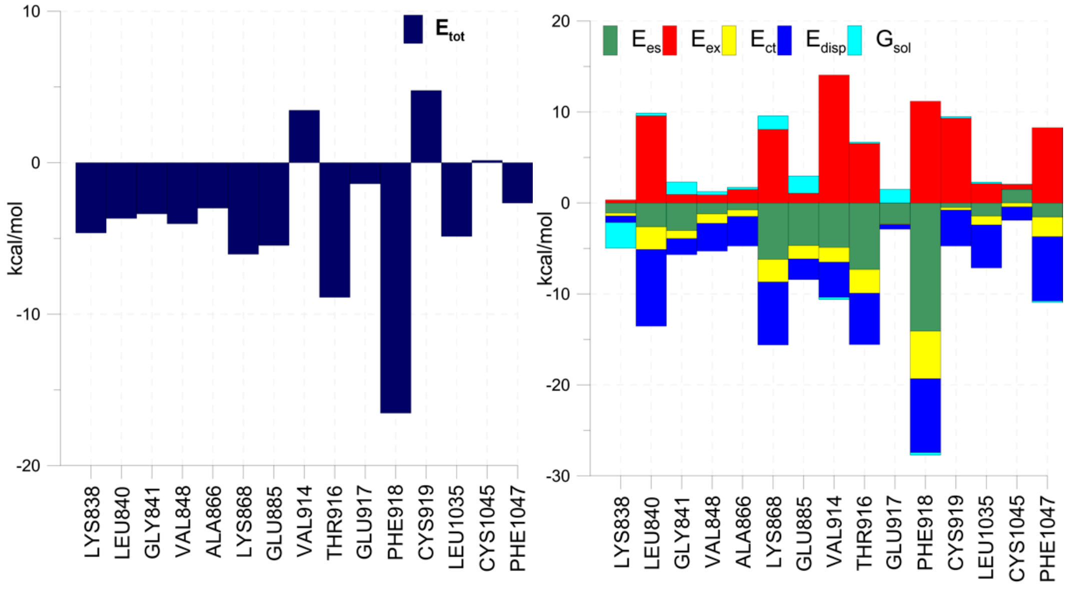

Fig. S8 Calculated interaction energies (E_{tot}; kcal/mol) and the contributions to the total energy (E_{es}, E_{ex}, E_{ct} + mix, E_{dis}, G_{sol}; kcal/mol) between docked azole **5** and selected residues of 3ewh.pdb kinase(*GAMESS* program).

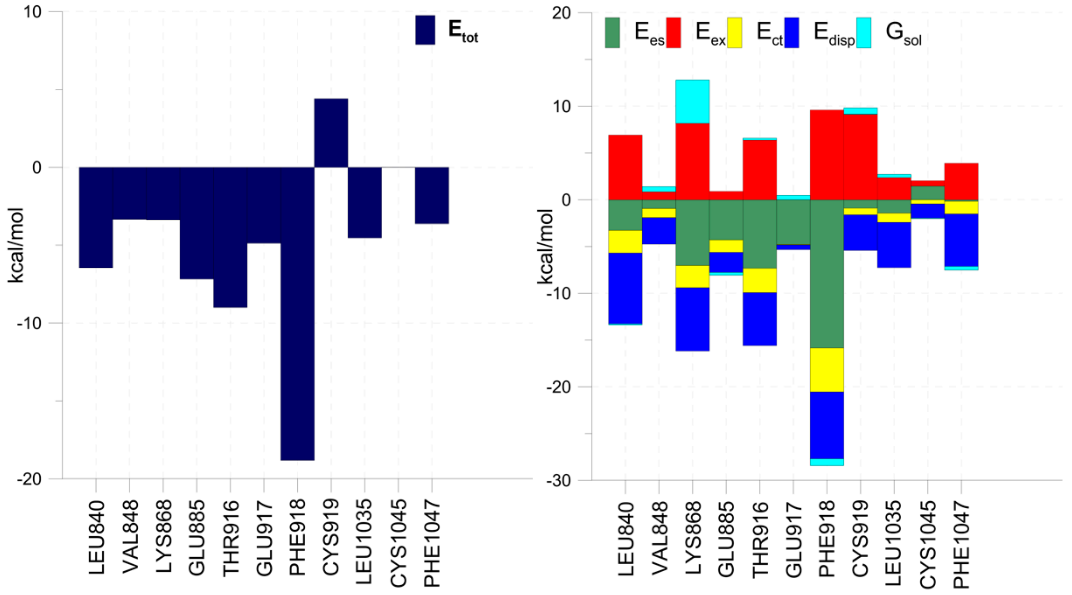

Fig. S9 Calculated interaction energies (E_{tot}; kcal/mol) and the contributions to the total energy (E_{es}, E_{ex}, E_{ct} + mix, E_{dis}, G_{sol}; kcal/mol) between docked azole **7** and selected residues of 3ewh.pdb kinase(*GAMESS* program).

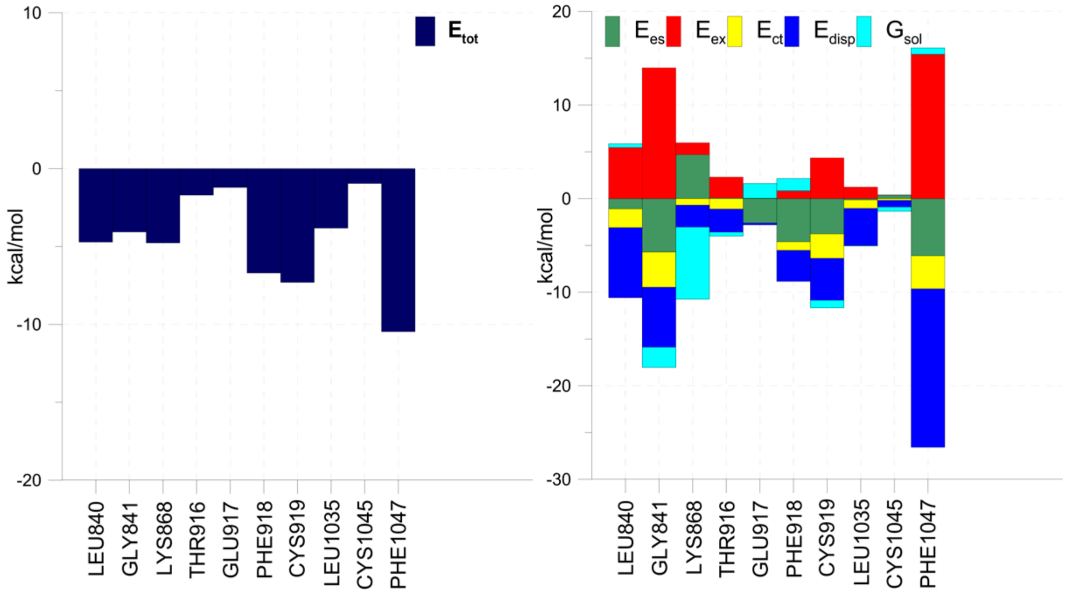

Fig. S10 Calculated interaction energies (E_{tot}; kcal/mol) and the contributions to the total energy (E_{es}, E_{ex}, E_{ct} + mix, E_{dis}, G_{sol}; kcal/mol) between docked azole **8** and selected residues of 3ewh.pdb kinase(*GAMESS* program).

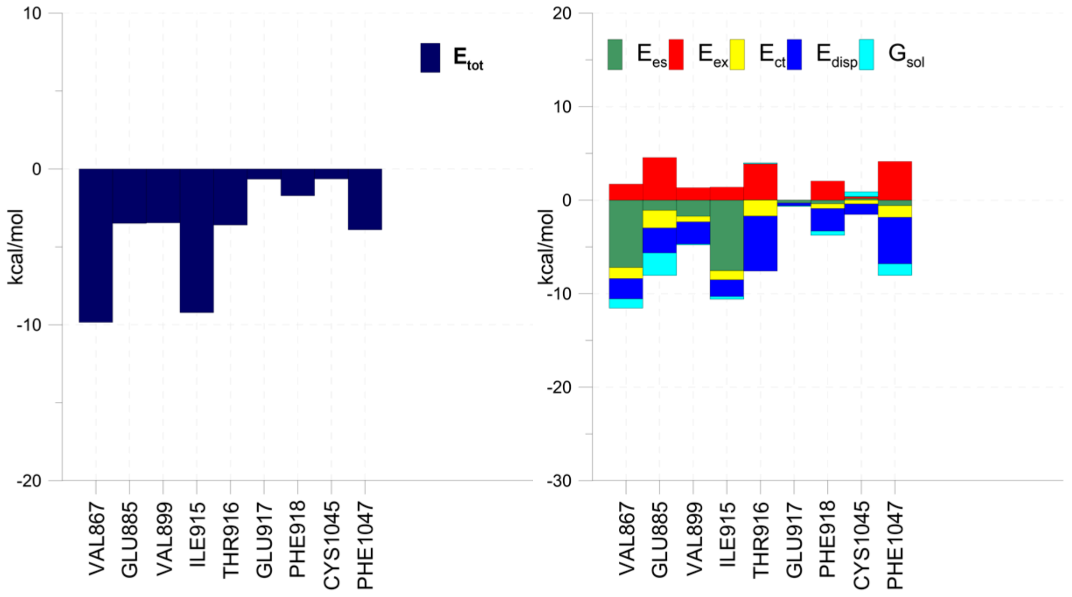

Fig. S11 Calculated interaction energies (E_{tot}; kcal/mol) and the contributions to the total energy (E_{es}, E_{ex}, E_{ct} + mix, E_{dis}, G_{sol}; kcal/mol) between docked azole **9** and selected residues of 3ewh.pdb kinase(*GAMESS* program).

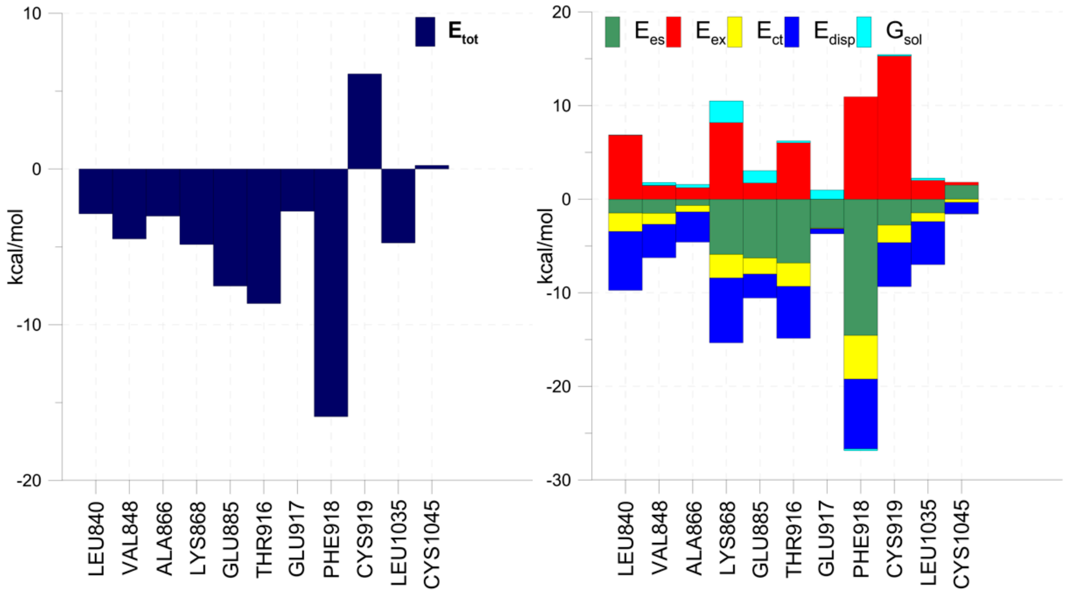

Fig. S12 Example of input file for SAPt calculations regarding the **1**–Glu917 complex:

```
memory 12 Gb

molecule {
0 1
N      13.09300     -12.93400      9.83300
H      12.37400     -12.58900     10.46800
C      12.68400     -13.52800      8.56300
H      13.03400     -14.58600      8.53500
C      13.30300     -12.77600      7.38300
O      13.43000     -11.55100      7.41100
C      11.16200     -13.51300      8.43700
H      10.83100     -14.12700      7.56700
H      10.69100     -14.09500      9.26300
C      10.57300     -12.10200      8.36200
H      11.10600     -11.49200      7.59600
H      10.83000     -11.51800      9.27600
C       9.07900     -12.08400      8.11700
O       8.59300     -12.87700      7.28200
O       8.37100     -11.22400      8.77700
H      14.07100     -12.86600     10.07800
H       7.84600     -10.58600      9.26700
O      13.69900     -13.43600      6.34100
H      13.99200     -13.92500      5.56900
--
C      16.05100      -6.98600      7.72300
C      16.26700      -7.42200      6.37600
C      15.46400      -8.58500      6.27300
N      14.86100      -8.73500      7.49600
N      15.21700      -7.77700      8.37400
C      15.38700      -9.33800      5.09200
C      16.13700      -8.89800      4.01500
C      16.94000      -7.73700      4.11600
C      17.02800      -6.98700      5.27500
H      14.22700      -9.45700      7.79700
Cl     17.85100      -7.25100      2.71800
S      16.78200      -5.57900      8.55700
O      15.77300      -4.51100      8.45800
O      18.14500      -5.39400      8.04500
C      16.89600      -6.08000     10.27500
C      16.05300      -5.49000     11.21600
C      16.15400      -5.87800     12.55100
C      17.08400      -6.84500     12.95600
C      17.20900      -7.23700     14.40700
C      17.91900      -7.42000     11.98500
C      17.83600      -7.04400     10.64800
H      14.77000     -10.22200      5.02600
H      16.10900      -9.44800      3.08600
H      17.65200      -6.10800      5.33200
```

```
H        15.33300      -4.74400      10.91500
H        15.50400      -5.42600      13.28600
H        18.63900      -8.16800      12.28100
H        18.48700      -7.49000       9.91100
H        16.47800      -6.68400      14.99700
H        18.21300      -7.00400      14.76100
H        17.02600      -8.30600      14.51100

units angstrom
}

set globals {
   basis         jun-cc-pvdz
   df_basis_scf  jun-cc-pvdz-jkfit
   df_basis_mp2  jun-cc-pvdz-ri
   guess         sad
   scf_type      df
}

set sapt { print 1 }

energy('sapt0')
```

Fig. S13 The protein–ligand interactions (**a;** hydrogen bonds – green, hydrophobic – white purple, ionic – pink, water bridges – blue) and schematic of detailed ligand atom interactions (**b**) for chlorine derivative **1**.

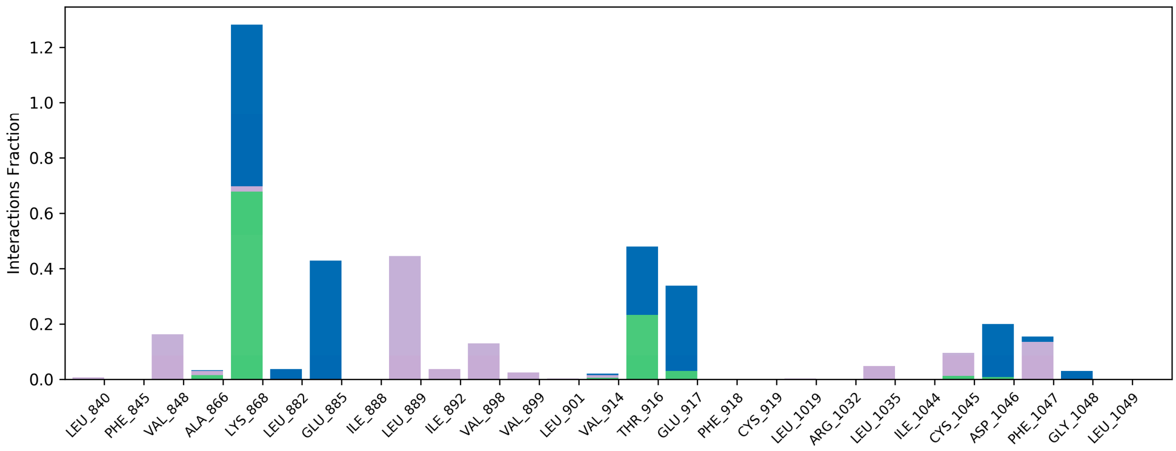

a

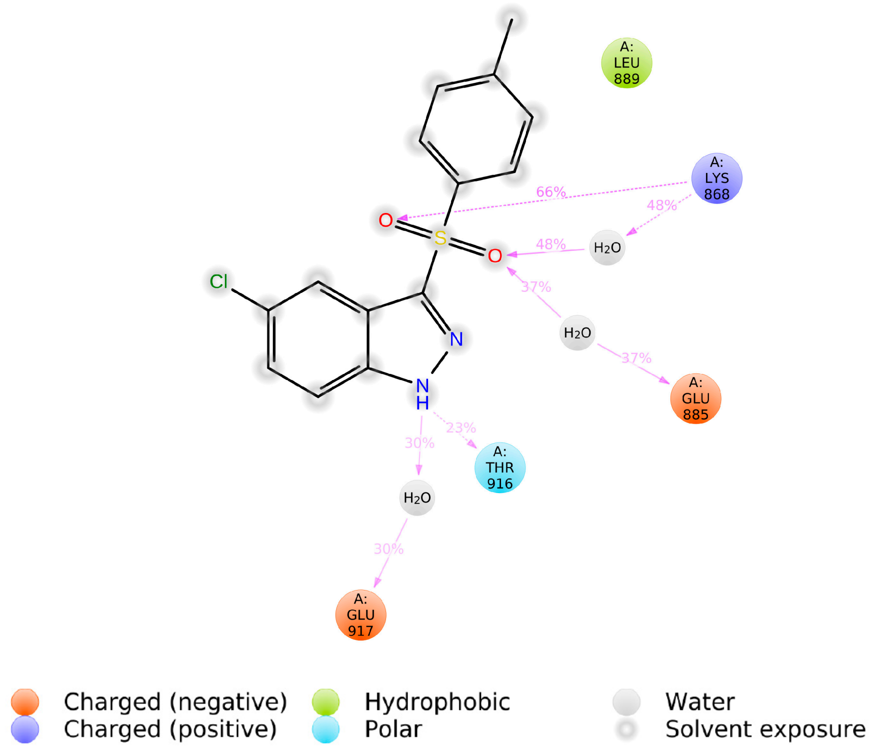

b

Fig. S14 The protein–ligand interactions (**a;** hydrogen bonds – green, hydrophobic – white purple, ionic – pink, water bridges – blue) and schematic of detailed ligand atom interactions (**b**) for pyrrole derivative **2**.

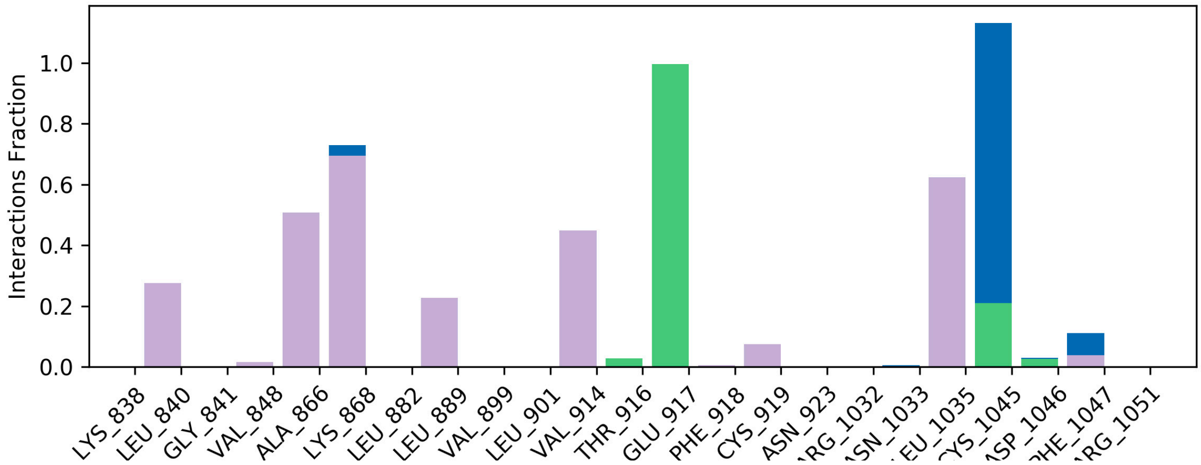

a

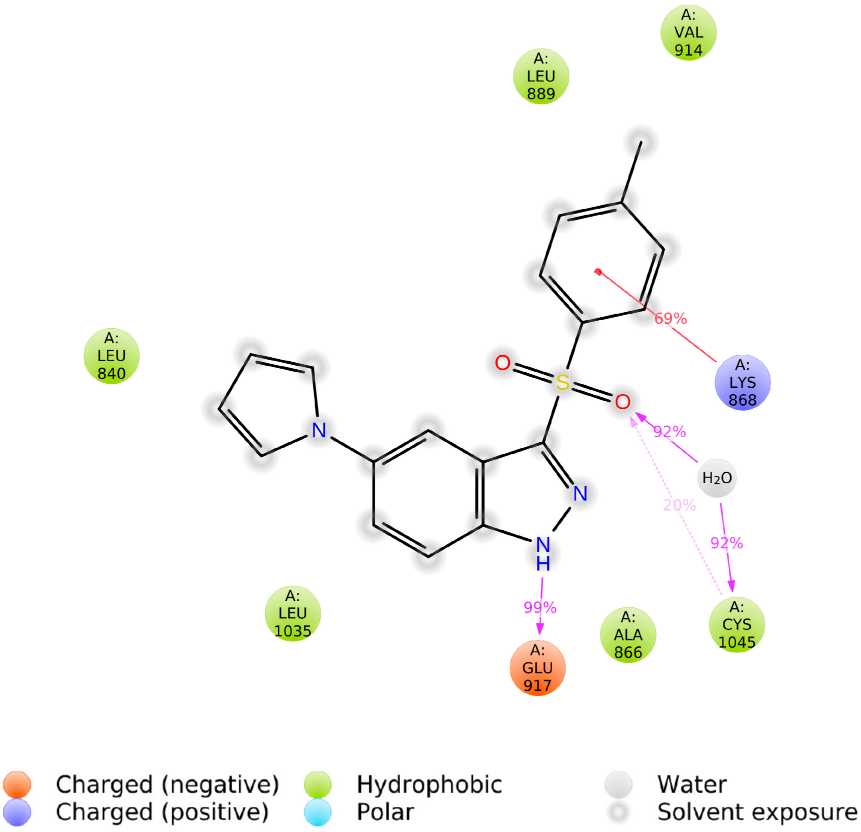

b

Fig. S15 The protein–ligand interactions (**a;** hydrogen bonds – green, hydrophobic – white purple, ionic – pink, water bridges – blue) and schematic of detailed ligand atom interactions (**b**) for pyrazole derivative **3**.

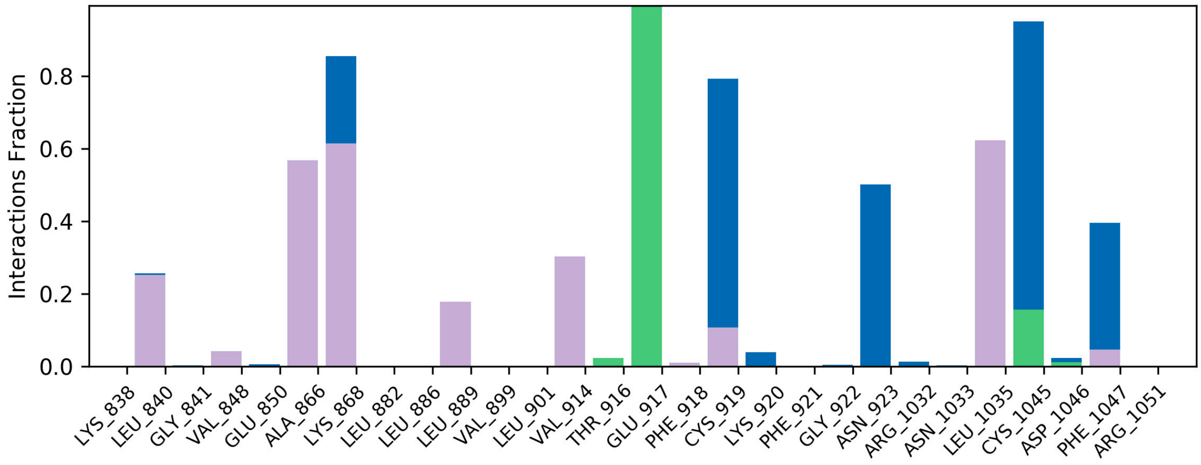

a

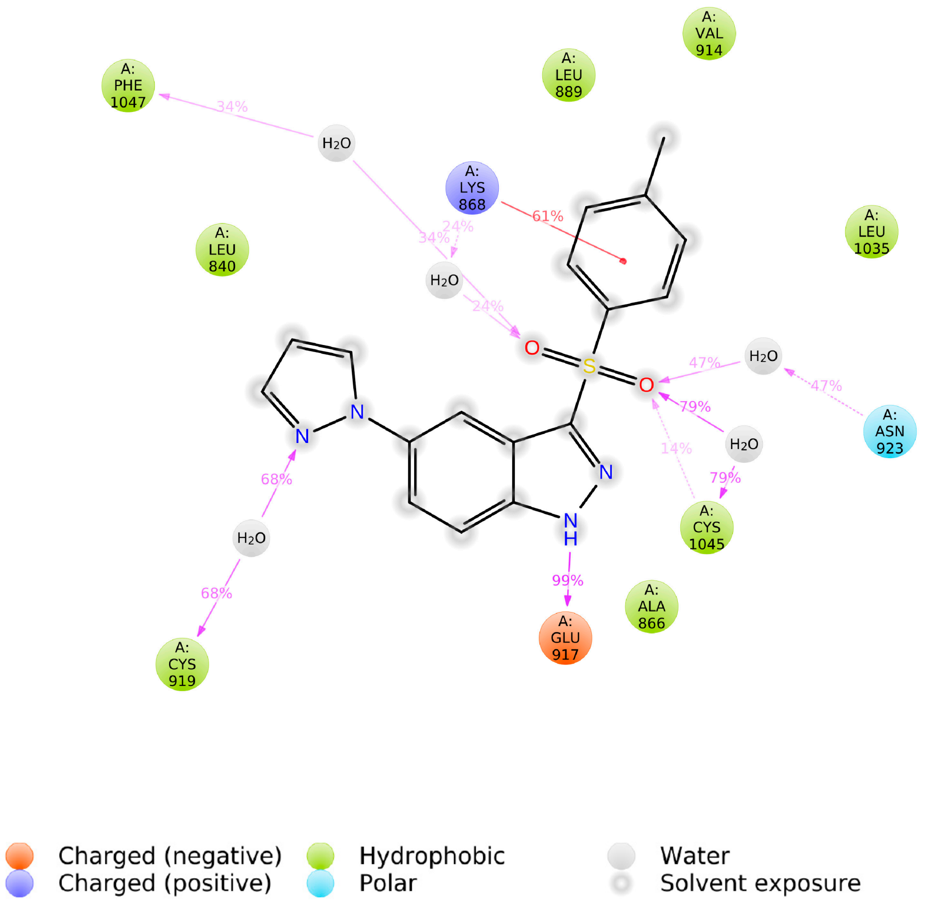

b

Fig. S16 The protein–ligand interactions (**a;** hydrogen bonds – green, hydrophobic – white purple, ionic – pink, water bridges – blue) and schematic of detailed ligand atom interactions (**b**) for dimethyl pyrazole derivative **4**.

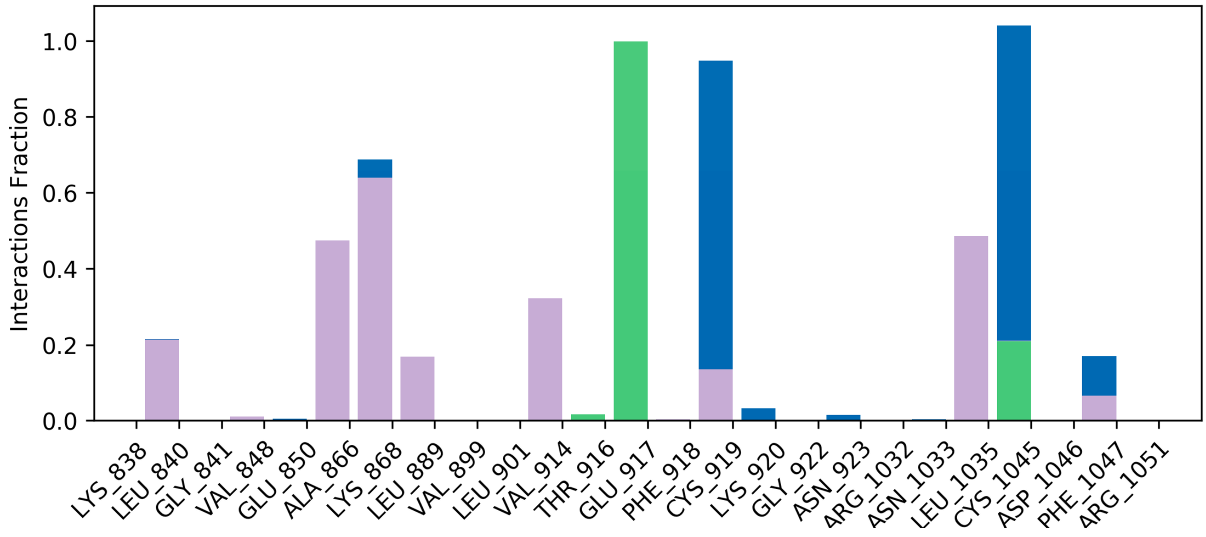

a

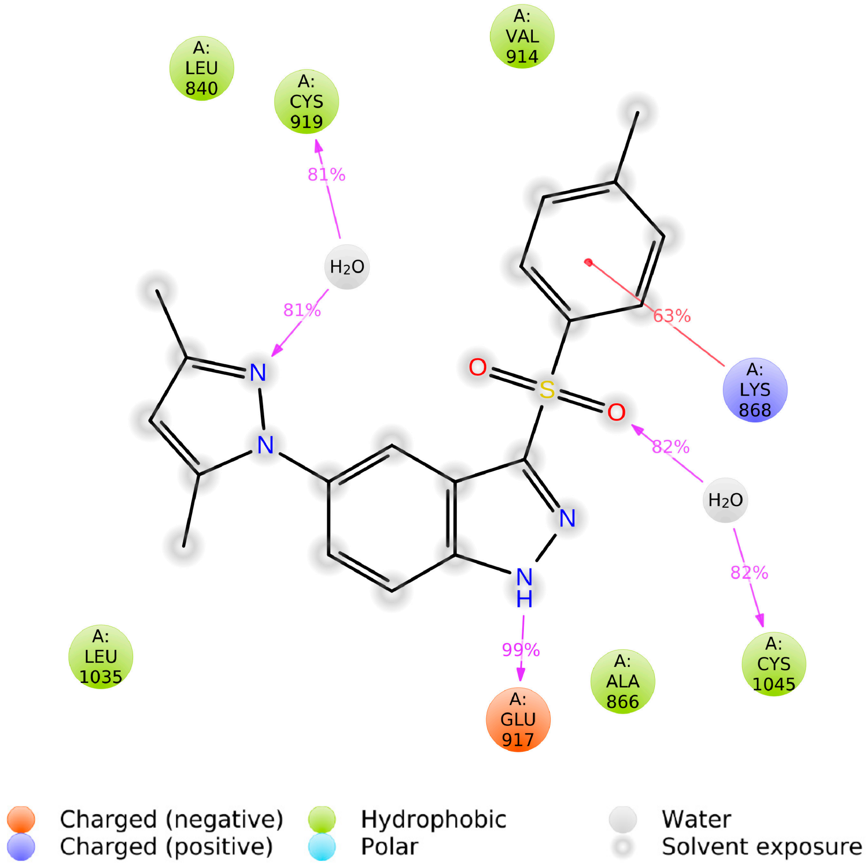

b

Fig. S17 The protein–ligand interactions (**a;** hydrogen bonds – green, hydrophobic – white purple, ionic – pink, water bridges – blue) and schematic of detailed ligand atom interactions (**b**) for triazole derivative **5**.

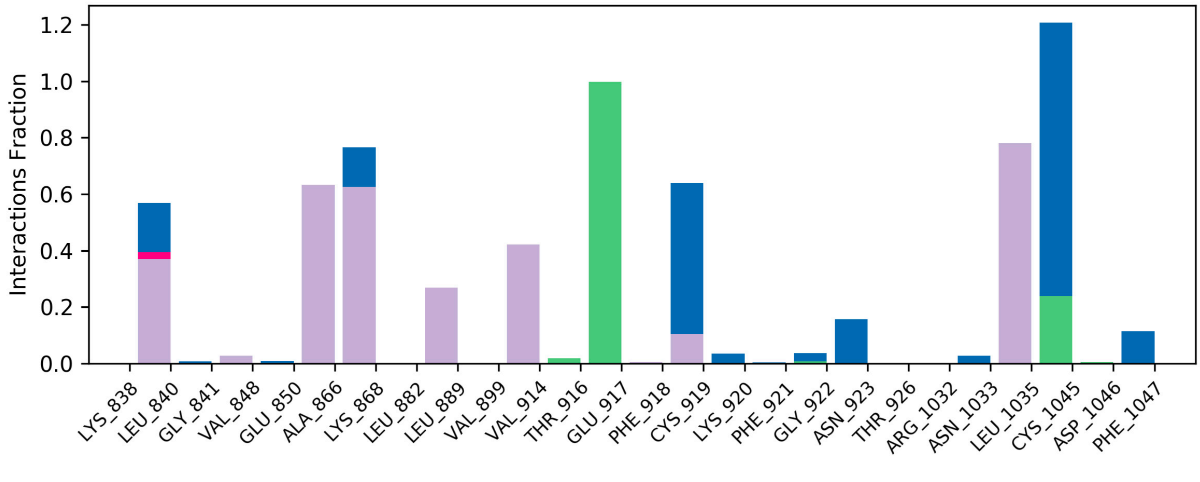

a

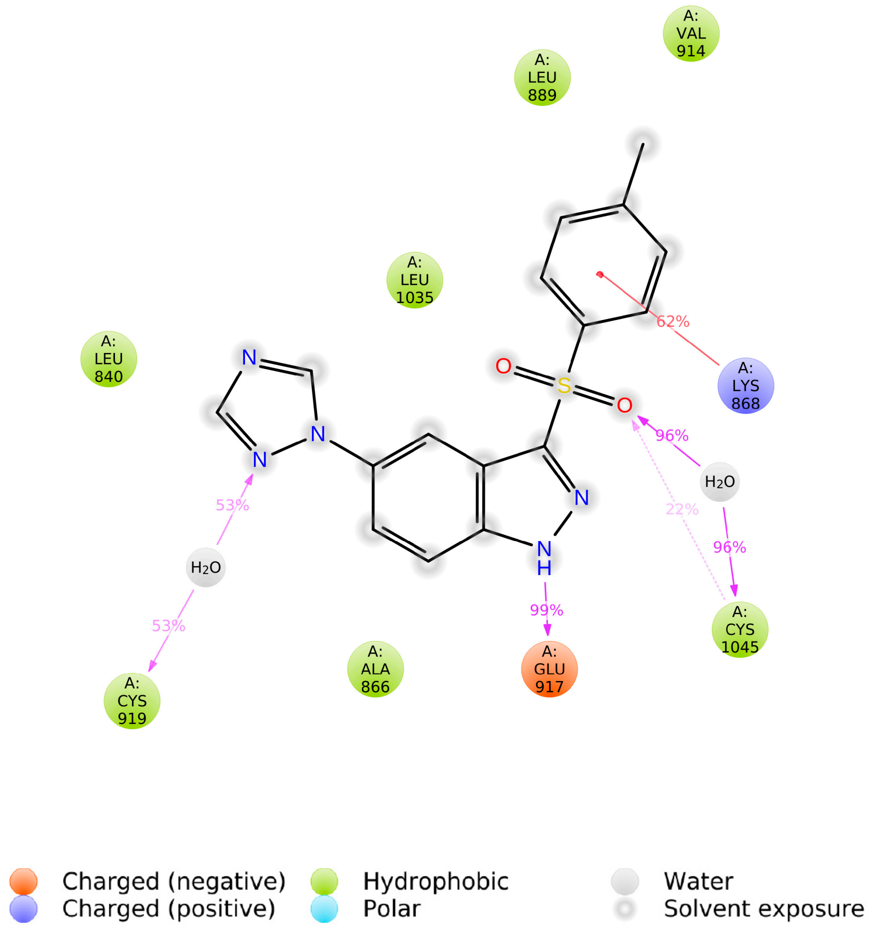

b

Fig. S18 The protein–ligand interactions (**a;** hydrogen bonds – green, hydrophobic – white purple, ionic – pink, water bridges – blue) and schematic of detailed ligand atom interactions (**b**) for indole derivative **6**.

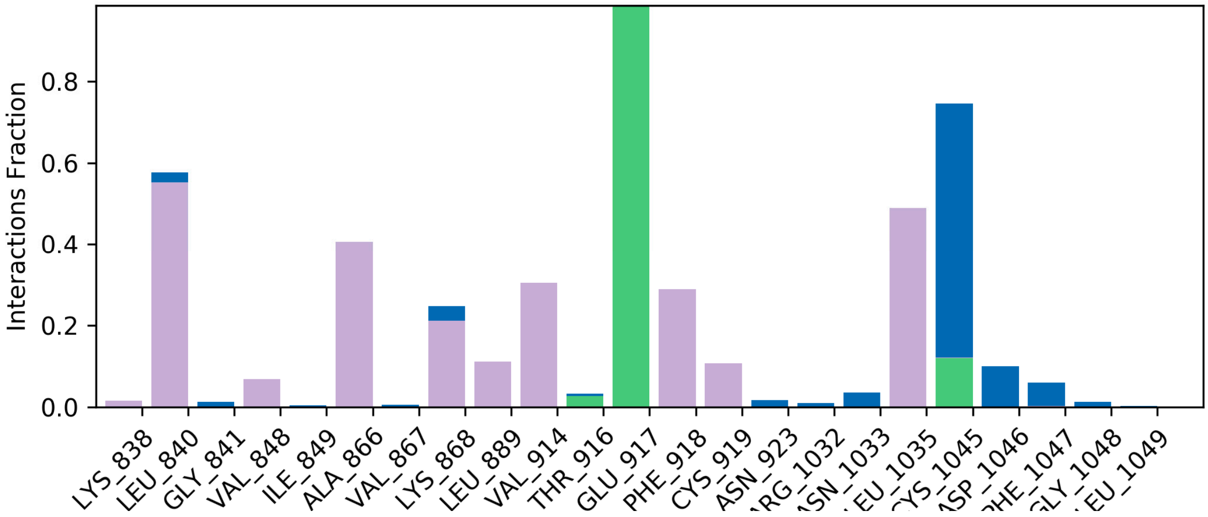

a

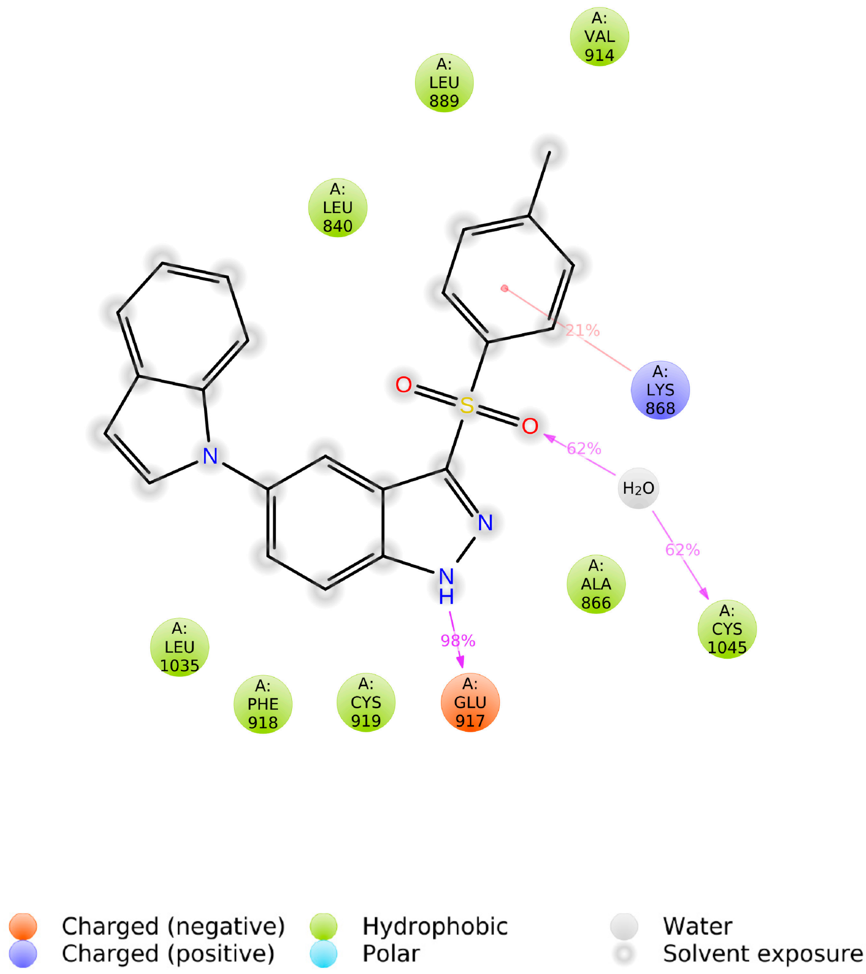

b

Fig. S19 The protein–ligand interactions (**a;** hydrogen bonds – green, hydrophobic – white purple, ionic – pink, water bridges – blue) and schematic of detailed ligand atom interactions (**b**) for carbazole derivative **8**.

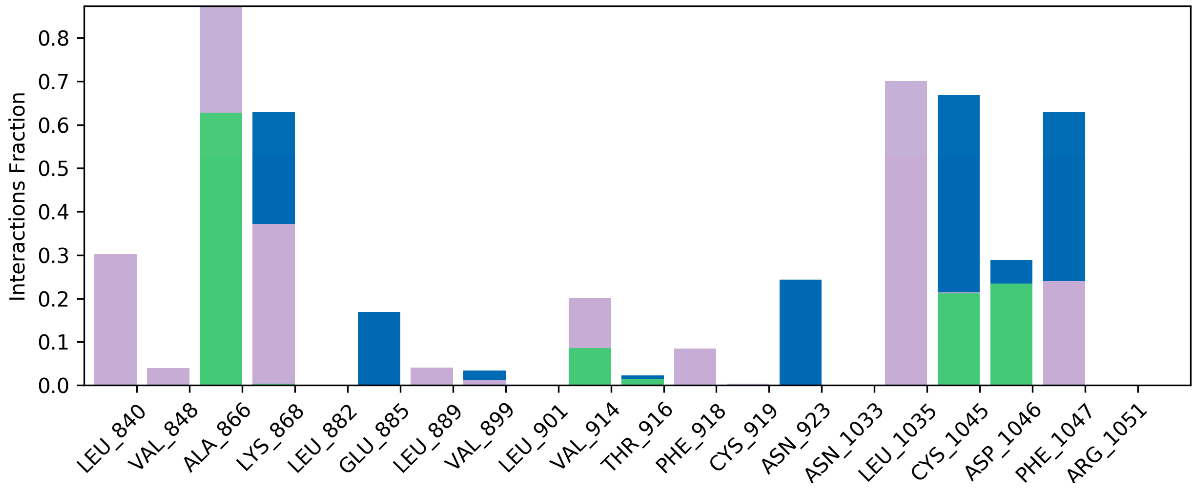

a

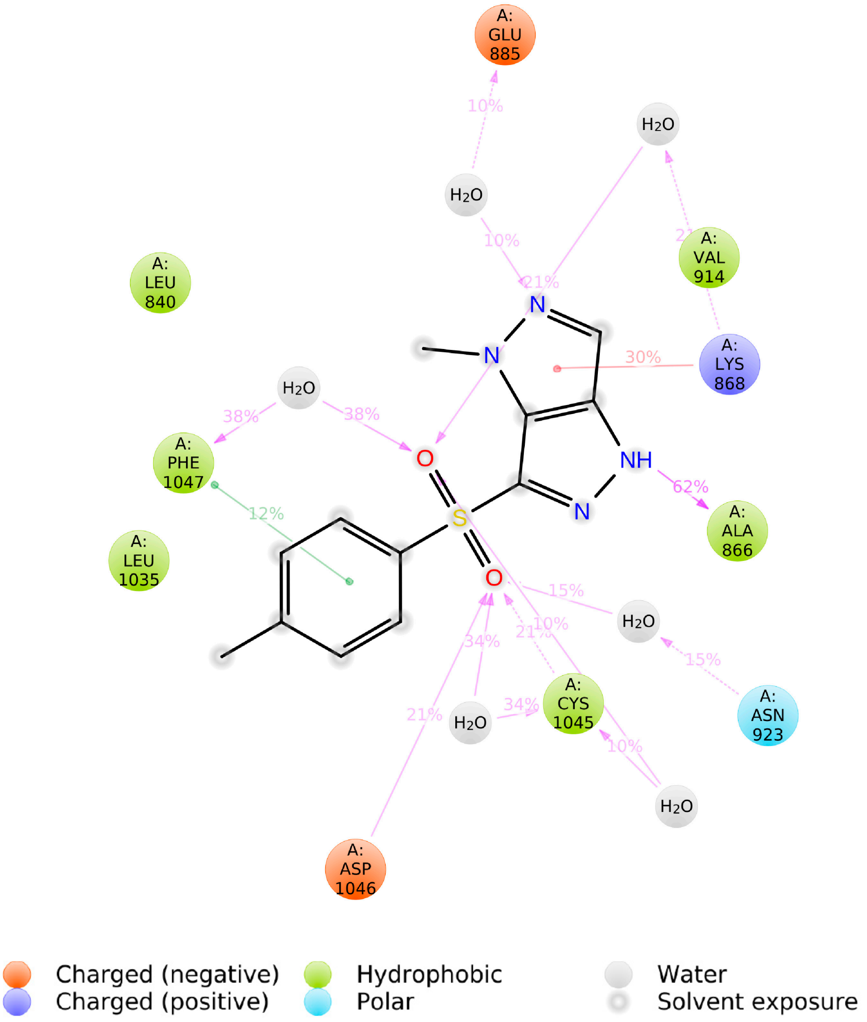

b

Fig. S20 Atoms numbering for RMSF calculations of the ligand **1** and the RMSF plot for **1** within ligand–protein complex during the MD productive phase calculated complex of kinase with **1**.

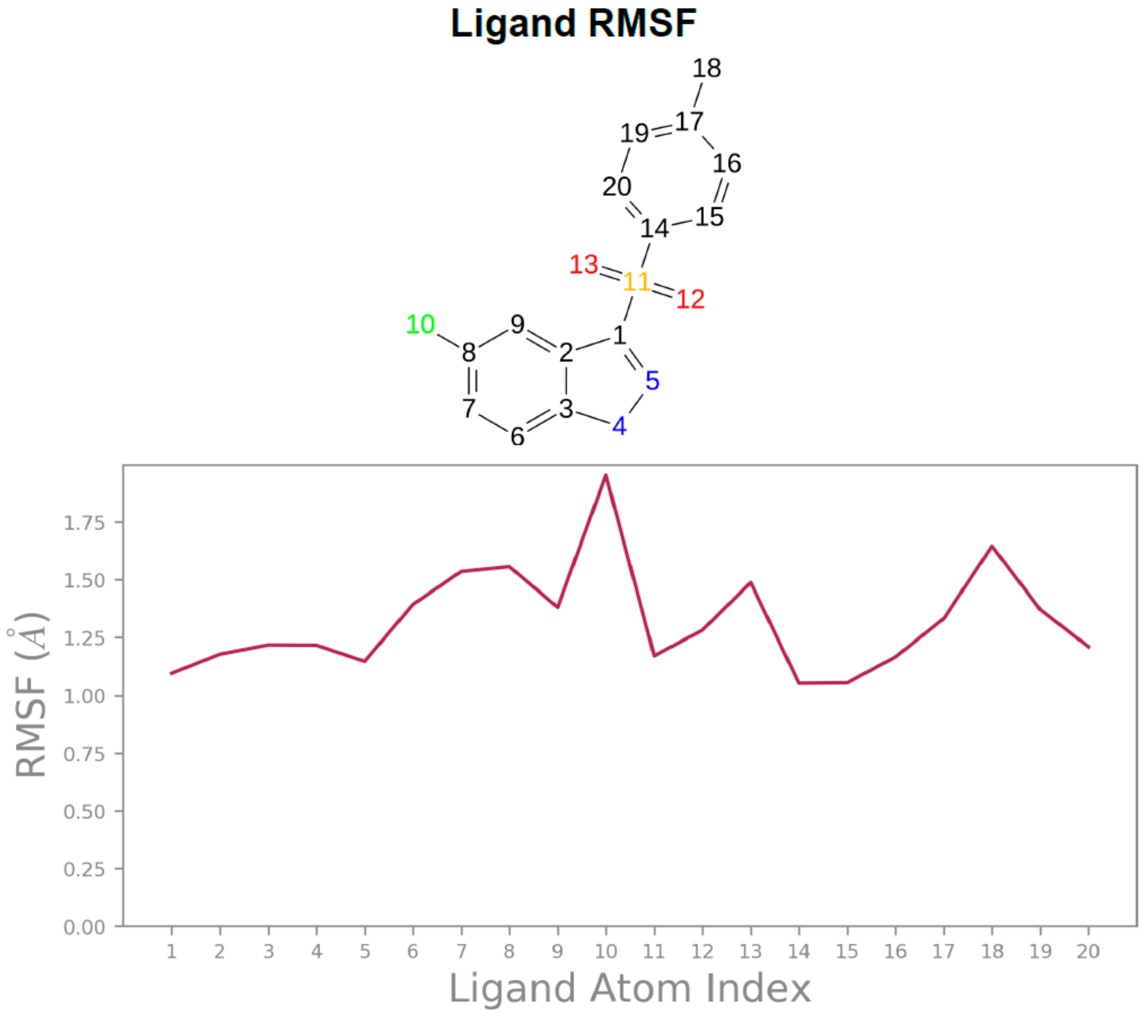

Fig. S21 The RMSD plot for the backbone within ligand-protein complex during the MD productive phase calculated complex of kinase with: **1–9** (*Y*-axis in Å); colors related with **Fig. 4** given in the manuscript.

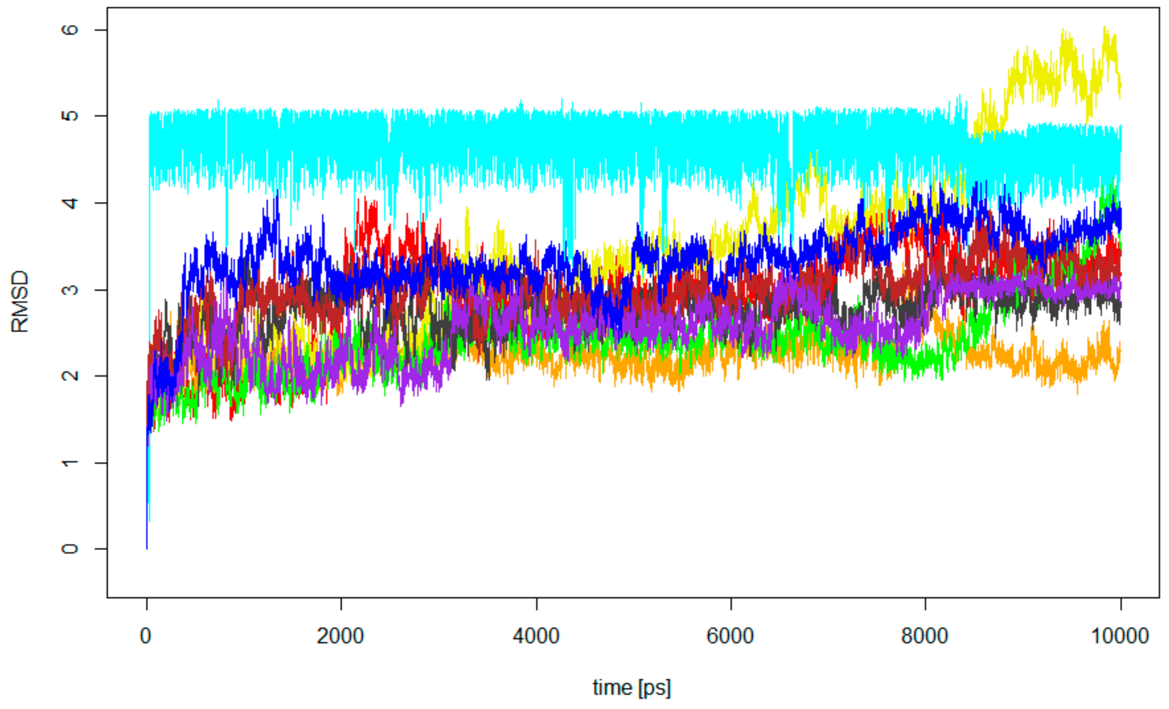

Fig. S22 Representative MD cluster for binding mode of **1** within the **1**–3ewh complex (**A**) and its comparison with results using docking protocol (**B**).

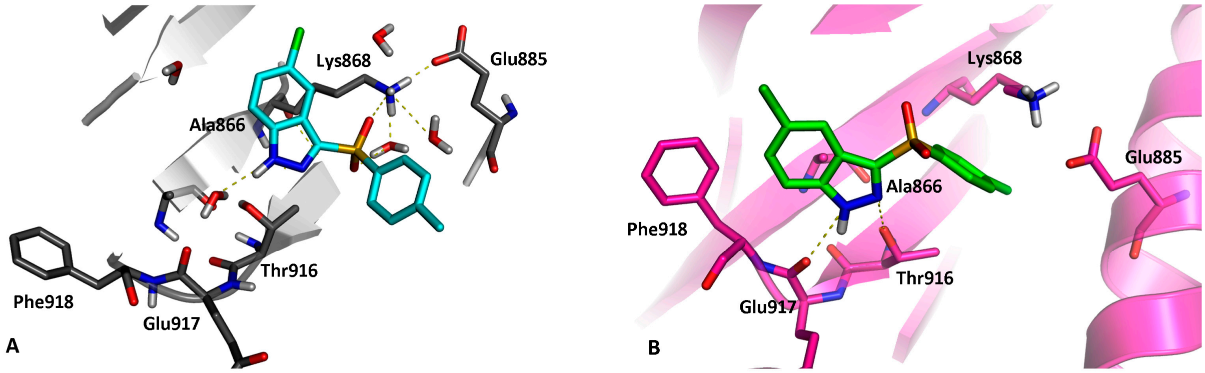

Fig. S23 Hydrogen contacts with Thr916 during the MD productive phase calculated complex of kinase with derivative **3**; red – angles [°], blue – distances [Å].

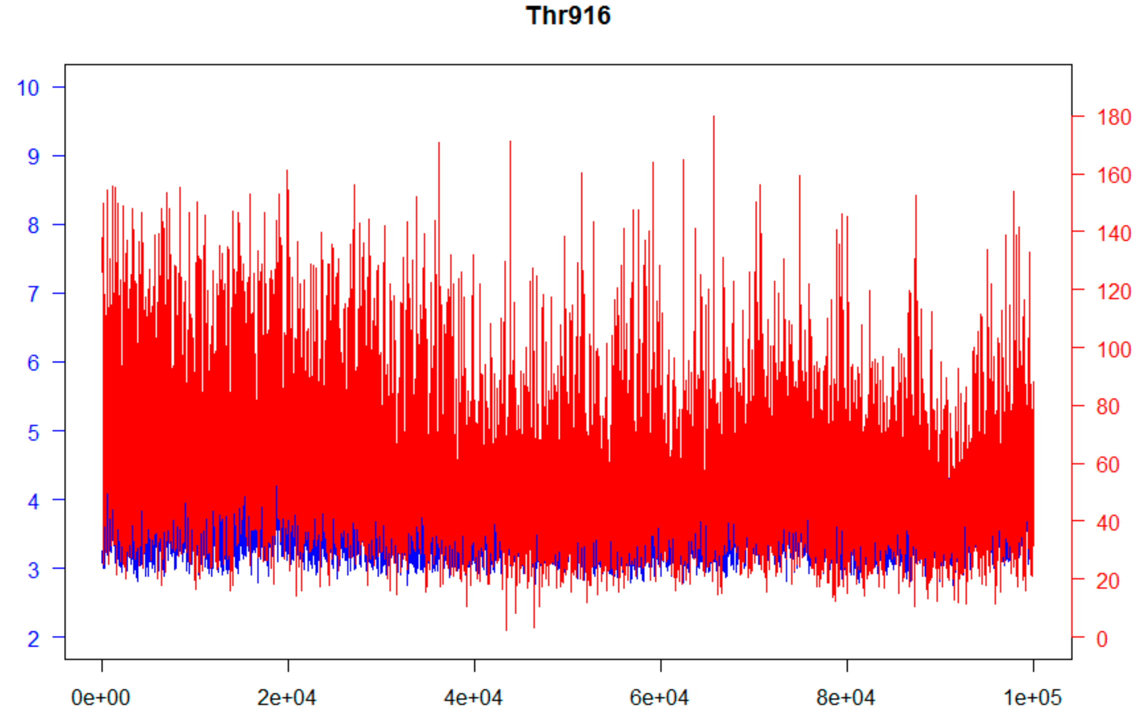

Fig. S24 Hydrogen contacts with Glu917 during the MD productive phase calculated complex of kinase with derivative **3**; red – angles [°], blue – distances [Å].

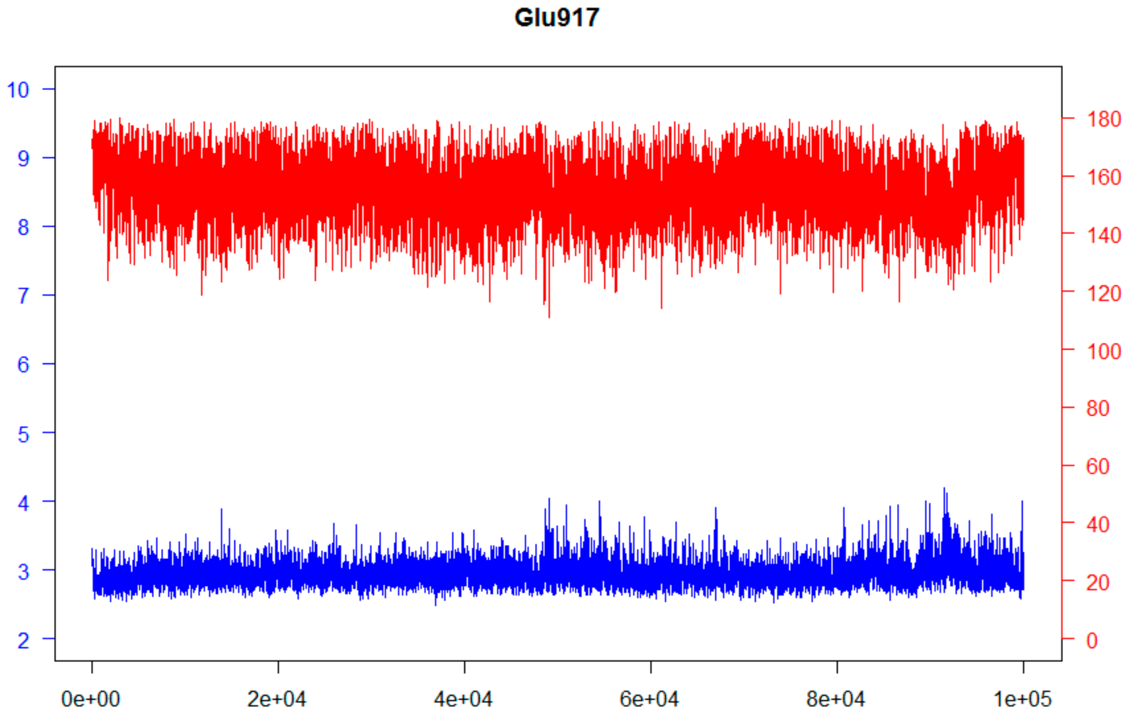

Fig. S25 Hydrogen contacts with Thr916 during the MD productive phase calculated complex of kinase with derivative **6**; red – angles [°], blue – distances [Å].

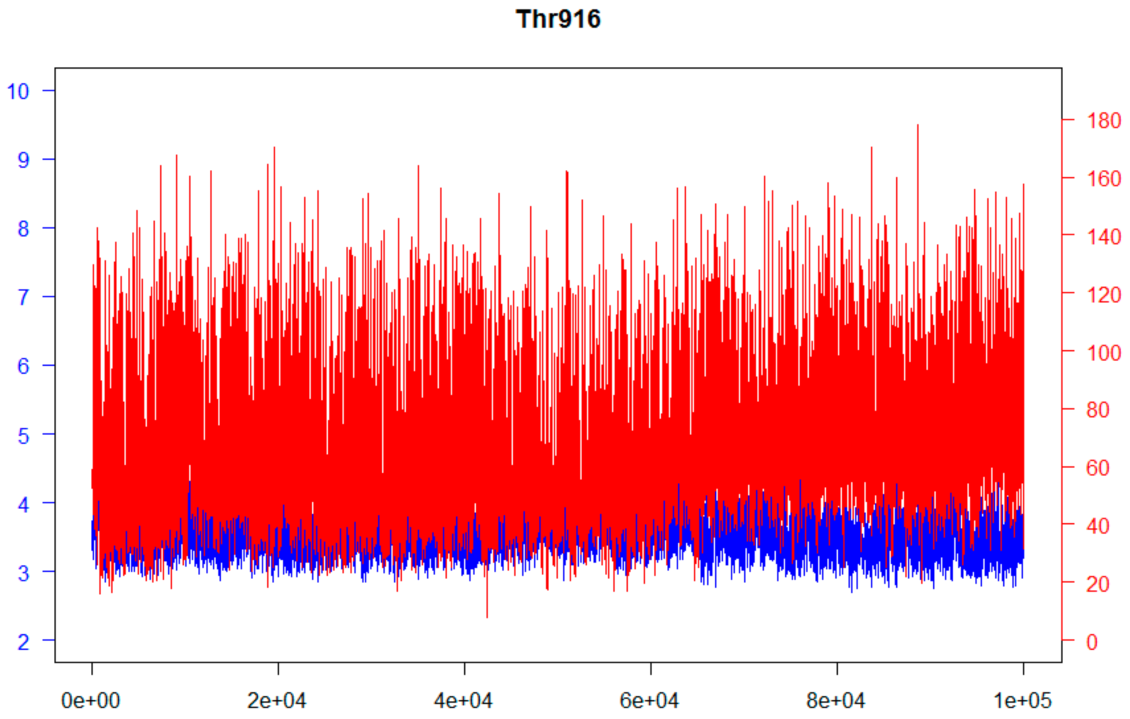

Fig. S26 Hydrogen contacts with Glu917 during the MD productive phase calculated complex of kinase with derivative **6**; red – angles [°], blue – distances [Å].

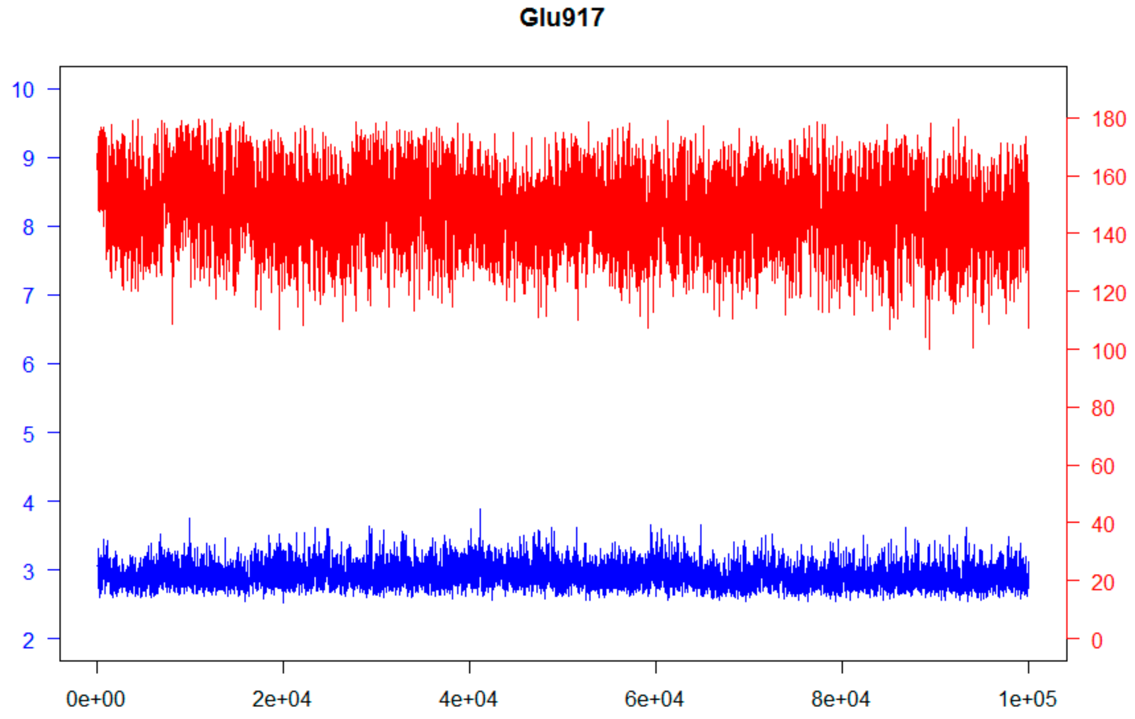

Supplement: Supplementary file 1 [file ijms-21-04793-s001.pdf]
